# RNomics and Modomics in the halophilic archaea *Haloferax volcanii*: identification of RNA modification genes

**DOI:** 10.1186/1471-2164-9-470

**Published:** 2008-10-09

**Authors:** Henri Grosjean, Christine Gaspin, Christian Marck, Wayne A Decatur, Valérie de Crécy-Lagard

**Affiliations:** 1 Department of Microbiology and Department of Microbiology and Cell Science, University of Florida, Gainsville, FL-32611, Florida, USA; 2 IGM, Université de Paris-sud, UMR 8621, Orsay, F 91405, France; 3 CNRS, IGM, Orsay, F-91405, France; 4 National Institute of Agronomical Research, Biometrics and Artificial Intelligence Department, Chemin de Borde-Rouge, Auzeville BP 27, 31326 Castanet-Tolosan, France; 5 Institut de Biologie et de Technologies de Saclay (iBiTecS), Commissariat à l'Energie Atomique (CEA), Gif sur Yvette, F-91191, France; 6 Department of Biochemistry and Molecular Biology, University of Massachussets, Amerherst, MA 01003, Massachusetts, USA

## Abstract

**Background:**

Naturally occurring RNAs contain numerous enzymatically altered nucleosides. Differences in RNA populations (RNomics) and pattern of RNA modifications (Modomics) depends on the organism analyzed and are two of the criteria that distinguish the three kingdoms of life. If the genomic sequences of the RNA molecules can be derived from whole genome sequence information, the modification profile cannot and requires or direct sequencing of the RNAs or predictive methods base on the presence or absence of the modifications genes.

**Results:**

By employing a comparative genomics approach, we predicted almost all of the genes coding for the t+rRNA modification enzymes in the mesophilic moderate halophile *Haloferax volcanii*. These encode both guide RNAs and enzymes. Some are orthologous to previously identified genes in Archaea, Bacteria or in *Saccharomyces cerevisiae*, but several are original predictions.

**Conclusion:**

The number of modifications in t+rRNAs in the halophilic archaeon is surprisingly low when compared with other Archaea or Bacteria, particularly the hyperthermophilic organisms. This may result from the specific lifestyle of halophiles that require high intracellular salt concentration for survival. This salt content could allow RNA to maintain its functional structural integrity with fewer modifications. We predict that the few modifications present must be particularly important for decoding, accuracy of translation or are modifications that cannot be functionally replaced by the electrostatic interactions provided by the surrounding salt-ions. This analysis also guides future experimental validation work aiming to complete the understanding of the function of RNA modifications in Archaeal translation.

## Background

Post-transcriptional modification of transfer and ribosomal RNAs is essential for their cellular activities as core molecules of the translation apparatus. To date, the chemical structure of more than one hundred RNA modifications have been identified in all domains of life [1-3]. In transfer RNAs, modified nucleotides are found predominantly within the 3D-core of molecules and in the anticodon arm, especially at the wobble position 34 and at position 37, 3' adjacent to the anticodon (conventional numbering of tRNA positions is as defined in [4], ). These particular modifications allow the molecules to adopt the canonical L-shaped conformation and modulate interactions with various interacting macromolecules such as aminoacyl:tRNA-synthetases, initiation, elongation and termination factors, mRNA and/or elements of the decoding and peptidyl-centers of the ribosome (reviewed in [5-10]). In ribosomal RNAs, modified nucleotides are located mostly in regions corresponding to the functional centers of the ribosome [11-14]. Their location suggests a role in accuracy and efficiency of translation, however the specific function of each modified nucleoside is still largely unknown. This lack of knowledge stems from peculiarities of the rRNA molecule itself: it is a large molecule (molecular mass between 1.5 to 3.9 MDa); some nucleotides are only partially modified and their function(s) are most certainly dependent on a network of synergistic interactions with different elements of the ribosome, including other modified nucleosides that may act cooperatively. Nevertheless, function has been attributed to modified nucleosides in rRNA in a few cases [13,15-22].

Difference in profile and type of RNA modifications (Modomics) is one of the criteria that distinguish the three kingdoms of life. While universal modifications such as Ψ, m^5^U, t^6^A or m^1^G are found in a large numbers of archaeal, bacterial and eukaryal tRNAs, each kingdom has a set of signature modifications. For examples mimG, G^+^, m^2^_2_Gm, ac^6^A or m^1^Ψ are typical of archaeal tRNAs, while yW, mcm^5^U and manQ, or k^2^C, mo^5^U and m^6^t^6^A are typical of tRNAs from Eukarya or Bacteria respectively (for review see Figure 8.1 in [23]). The same conclusion applies for modified nucleotides in rRNAs (see [24]; ).

In Archaea, our knowledge of the diversity of RNA modifications is largely founded on the lifework of Jim McCloskey and Pamela Crain, who analyzed bulk tRNA and rRNA preparations from a phylogenetically diverse set of Archaea. The technique used combined separation of nucleosides of bulk RNA RNase hydrolysate by liquid chromatography, followed by comparison of the derived modified nucleosides to synthetic ones by mass spectrometry techniques [25] (for more recent development of the technique, see [26] and references therein). However, to date *Haloferax volcanii*, a Halobacteriaceae that lives optimally at 42°C in the presence of 1.5–2.5 M NaCl [27], is the only Archaea for which both the chemical identities and positions of almost all modified ribonucleosides have been mapped for nearly the whole set of the 52 sequenced tRNAs with distinct anticodons [28,29]. In addition, 13 tRNA sequences of two closely related mesophilic halophiles are available, *Halobacterium cutirubrum *(12 sequences) and *Halococcus morrhuae *(one sequence) [4]. For *H. volcanii *ribosomal RNAs, the type and position of modifications are available in the case of the 16S RNA [30,31], but not for the 23S nor the 5S RNAs. These can be inferred from studies on another closely phylogenetically related halophilic Archaea *Haloarcula marismortui *[32,33]. However, while the RNA modifications have been mapped in RNAs of halophiles, including *H. volcanii*, the identity of the genes that code for the corresponding RNA modification enzymes remains largely ignored.

Using a comparative genomic analysis method, that we have recently applied to the only other organism with an almost complete set of sequenced tRNAs, the pathogenic bacteria *Mycoplasma capricolum *[34], we set out to predict all the RNA modification genes in the halophilic archaeaon *H. volcanii*. Some were easily predicted by homology with experimentally validated RNA modification genes from other organisms, while a few are original predictions based on comparative genomic analysis [35] (not based on homology). This computation work provides predictions that can now guide the experimental validation work with the goals of elucidating the role of RNA modifications in Archaeal translation, and ultimately obtaining a better understanding of the emergence of this extraordinary complex enzymatic machinery during evolution.

## Results and Discussion

### Post-transcriptional modification of RNA

#### Modification pattern of tRNAs

**Figure 1:**
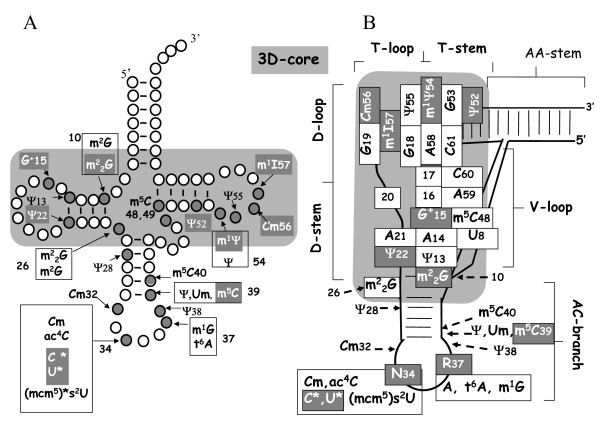
**Type and location of modified nucleosides in tRNAs of *H. volcanii*.(A) **2D-Cloverleaf representation of tRNA. Group of nucleosides in boxes are present at the same location but in different isoacceptors species. Modified nucleosides in white and in a gray background are uniquely present at that position in archaeal tRNAs so far. Those indicated in black are also found in tRNAs from Bacteria and/or Eukarya. See text for references on Abbreviations. The large gray box including the m^1^Ψ containing branch and the G^+ ^containing branch encompass the interacting parts of the tRNA molecule that forms the 3D-core. **(B) **Schematic representation of tertiary interactions in tRNA structure. Each nucleoside involved in stacking or base pairing with another nucleotide within the 3D-core (gray background box) is represented by a rectangle. Other parts of the tRNA (anticodon branch and amino acid stem are represented by lines. Inside the large gray rectangle are the elements that contribute to the 3D interaction, allowing an L-shaped spatial conformation to be formed from the 2D cloverleaf structure.

Thanks to the "tour de force" of Gupta [28,29], a list of almost all of the modified nucleosides present in the different tRNAs of *Haloferax volcanii*, a typical mesophilic halophile, is available. Figure 1A shows their distribution (identity and location) in the general 2D-cloverleaf structure of tRNA, while Figure 1B shows their positions in a schematic 3D-architecture model. The modifications that are unique to archaeal tRNAs are shown in gray. For example G^+^-15 (for Archaeosine), C*-34 (for a lysidine-type of nucleoside) and m^1^Ψ-54 are unique to all archaeal tRNAs analyzed so far, both by their chemical structure and their positions in the nucleic acid, while others such as m^2^_2_G-10, Ψ-22, Ψ-52, C_m_-56 and m^1^I-57 (I for inosine) are unique only because of their position, rather than their chemical structure (see in [1,4], reviewed in [23]). Since tRNA modification enzymes are usually site-specific, we expect the corresponding genes to be different from those in the other kingdoms. In terms of chemical structure (and not their position), the modified nucleosides m^2^_2_G, m^5^C and m^1^I found in tRNAs of *H. volcanii *are characteristic of eukaryal rather than eubacterial tRNAs, while D (for dihydrouridine), I (for inosine), m^5^U, i^6^A (i for isopentenyl), Q (for queuosine) and m^7^G, which are common in tRNAs of Bacteria and Eukarya, are absent in *H. volcanii *(but not necessarily in other Archaea – see Fig 8.1 in [23]). Also, as mentioned in the Methods section, m^1^G at position 9 and m^5^C at positions 50–52 are found in tRNAs from another halophile, *H. cutirubrum*, but absent in these positions in all *H. volcanii *tRNAs.

As illustrated in Figure 1B, the modified nucleosides in tRNA can be classified in two categories: those that are present in the 3D-core (gray background) and presumably implicated mostly in the formation and/or the control of flexibility of the L-shaped molecule (reviewed in [9,36]); and those present in the decoding region (anticodon hairpin), implicated in the efficacy and accuracy of interaction with selected amino-acyl tRNA synthetases (reviewed in [7]) and the various codons within the mRNA:ribosome complex (reviewed in [5,8]).

The identification of a modified nucleotide does not imply it is present in a one to one ratio with the RNA molecule. Indeed, the presence and final chemical structure of certain modified nucleotides, particularly the hypermodified ones, may vary according to the physiological constraints of the cell (aerobic/anaerobic conditions, temperature, availability of intermediate metabolites or cofactors of the modification enzymes, various metabolic stress conditions; discussed in: [37-41]). The A-15, C-34, U-52 and U-54 residues in some *H. volcanii *tRNAs were reported to be only partially modified into G^+^-15, ac^4^C-34 (ac for acetyl), Ψ-52 or Ψ-54/m^1^Ψ-54 respectively, giving rise to distinct iso-tRNA species that sometimes can be separated by liquid chromatography or 2D-gel electrophoresis [28]. When a modification requires multiple modification enzymes like m^1^Ψ-54, G^+^-15 and few U-34 derivatives (see below), only intermediate products may exist under certain physiological conditions. However, the genes corresponding to all of the expected modified nucleotides (present or not) in the cellular tRNA population should be present in the genome.

#### Modification pattern of rRNA

**Figure 2:**
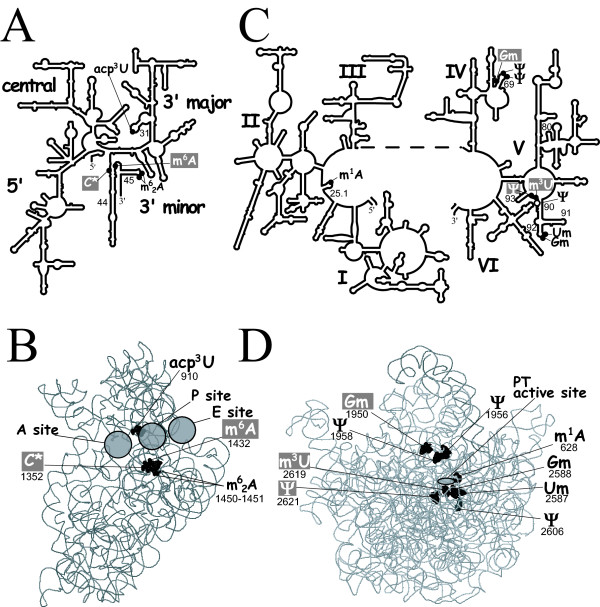
**Distribution in the ribosome of modified nucleosides in Halobacteriaceae rRNA.(A) **A schematic of secondary structure of *H. volcanii *16S rRNA with the locations of the various modified nucleosides indicated with darkened circles. The helices in which these appear are numbered according to the designations used for *E. coli ***(B) **The locations of modified nucleosides of *H. volcanii *16S rRNA are highlighted in a crystal structure of the small ribosomal subunit derived for *Thermus thermophilus *(PDB entry 2j00; portion of 70S) by showing full atomic volume (van der Waals radii) darkened on a backbone of the rRNA. The decoding center region is indicated by shading where the anti-codon stem loops of the A, P, and E-site tRNAs sit. **(C) **A schematic of secondary structure of *H. marismortui *23S rRNA with the locations of the various modified nucleosides indicated with darkened circles. **(D) **The locations of modified nucleosides of *H. marismortui *23S rRNA are highlighted in a crystal structure of the large ribosomal subunit derived for *Thermus thermophilus *as described above. The peptidyl transferase active site is indicated by shading where the acceptor stems of the A- and P-site tRNAs sit. The location of the Ψ residue of *H. salinarum *is highlighted with an open circle in (C) and lighter gray atomic volume in (D). In (B) and (D), the subunit interface is towards the front.

**Figure 3:**
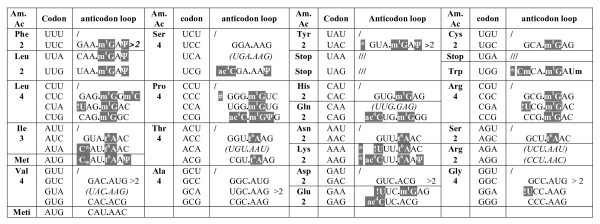
**Decoding strategy in *H. volcanii*.** The various sense codons of mRNA (from 5' to 3') are boxed according to their correspondence with one of the 20 amino acids. In each decoding box containing 1, 2, 3 or 4 synonymous codons are indicated the corresponding sequences of anticodon loop in tRNA (from nucleotide at the wobble position 34 to nucleotide at position 39, on the 3' side of the anticodon, the three first bases being the anticodon). A dash line means no tRNA with strictly complementary codon exists. The modified nucleotides are indicated in white under gray background. Abbreviations are the conventional ones as defined in [4] except for symbol C* in the case of one tRNA-Ile (C*AU) which correspond to a yet unknown modified cytosine at position 34. Likewise, symbol !U in the wobble position of several tRNAs correspond to a yet experimentally unidentified uridine derivative. In the case of tRNA-Gln, tRNA-Lys and tRNA-Glu, !U probably correspond to a mcm^5^s^2^U or a similar type of U-derivative (for details see text). Symbol * in front of a sequence means a Cm is present at position 32, while symbol # note the presence of an unexpected A instead of the usual pyrimidine C or U at position 32. No inosine has been found at the wobble position of any tRNA. The sequences indicated between brackets and in italics correspond to the tDNA sequence only. A number >2 on the right of the anticodon sequence means there exist 2 genes harboring the same anticodon on the genome. In all other cases, only one single gene exists (no redundancy). There is no tRNA-Sel/Sec coding for selenocysteine in *H. volcanii*. For more details see Additional files 1 and 2.

In their early work, Gupta and Woese identified four positions with modified bases in *H. volcanii *16S RNA[30]. These were later confirmed [31] and identified as acp^3^U-910 (position 966 by *E. coli *numbering; acp for 3-amino-3-carboxypropyl, thus an amino acid) in hairpin 31 located in the 3' major domain, m^6^A-1432 (position 1500) in helix 44, the tandem m^6^_2_A-1450 and m^6^_2_A-1451 (positions 1518 and 1519) in hairpin 45 and a modified cytidine (C*) of still unknown structure (MW:330.117 as determined by mass spectrometry) at position 1352 (1404) in helix 44 in the 3' minor domain of SSU RNA. Their locations are shown in the schematic 2D-structure (Figure 2A) and 3D-structure (Figure 2B) of 16S rRNA.

The characteristic pair of tandem dimethylated adenosine (m^6^_2_A m^6^_2_A) is universally present at analogous positions in rRNA of all organisms examined so far. These are located at the interface of the two ribosomal subunits [11,12,14] and their formation may serve as a checkpoint in quality control of ribosome biogenesis [42-44]. Likewise, acp^3^U-910 (966) in hairpin 31 appears to be nearly universally modified, although the type of base and corresponding modification vary from one organism to another: m^2^G 3'-adjacent to a m^5^C in 16S RNA of both *E. coli *and *Thermotoga maritima *[45], m^2^_2_G 3'-adjacent to m^5^C in *Thermus thermophilus *[46], m^1^Ψ-acp^3^U in *Drosophila melanogaster *SSU RNA and designated as unknown modified nucleoside in SSU RNA of other organisms, mostly archaeons [31]. This modified nucleotide is above the P-site-bound tRNA and directly contacts the anticodon stem-loop of tRNA at position 34 [47-51], and is also often modified (see Figure 2A and Additional file 1 ). Several studies indicate this nucleotide is important in decoding genetic information, particularly at the step of initiation [21,22,52].

Helix 44 is the dominant structural component of the 30S subunit interface. It's upper end lies just below where the mRNA transverses the subunit in the P site [53,54]. This portion forms a significant intersubunit bridge while at the same time is directly functionally important for efficient and accurate decoding since two bases, at least in the *E. coli *ribosome (bases 1492 and 1493) flip out of an internal loop in this region [53,54]. This allows the monitoring by direct contact of the mRNA-tRNA base pairing in the A site, a conformational transition facilitated by the binding of aminoglycoside antibiotic, e.g. paromomycin, to a pocket in the major grove of the top of helix 44 [55]. Modified nucleoside m^6^A-1432 (1500 *E. coli *numbering) at the bottom of helix 44 is present in SSU RNA of most (if not all) Archaea, and only a few Eukarya, but never in Bacteria (for references see [31]). It is also termed a 'decoding site nt' [49] because it is present in the functionally significant region of helix 44, adjacent to a critical intersubunit bridge (B2a). Contrary to the others above, the unknown N-330 (C*1352; 1404 *E. coli *numbering) is found in Archaea and in many Bacteria, but not in Eukarya. While it directly contacts paromomycin bound to helix 44, its function remains an enigma and its chemical structure remains to be elucidated.

#### Modification pattern of large subunit rRNAs

No data are available for *H. volcanii *23S RNA modifications. We therefore used the analysis performed in the closely related organism *Haloarcula marismortui *[32,33,56] that led to the identification of modified nucleotides at eight positions. Their locations in the generalized schematic 2D and 3D-stucture of 23S rRNA are shown in Figures 2C and 2D.

Three Ψ residues are present: two of which, Ψ-1956 and Ψ-1958, are located at universally conserved positions (1915 and 1917; *E. coli *numbering) in helix 69 loop of domain IV. The helix 69 stem-loop contacts A- and P-site tRNAs, contributes to bridge regions B2a and B2b of 23S rRNA, is involved in translation termination, contacts ribosome recycling factor, plays an active role in dissociation of subunits at the end of translation, and is important for subunit association [17,33,49,57-62]. Specifically, Ψ-1956 (1915) contacts the D stem of tRNA in the A site (positions 11 and 12) and Ψ-1958 (1917) is immediately adjacent to bridge B2a contacts, as well as direct contacts to A-site tRNA; they are important for the conformational flexibility of helix 69 and their loss affects subunit association, dissociation, and translation termination [17,58-61]. The peptidyl transferase center of the large ribosomal subunit is the rRNA that directly surrounds the active site of peptide bond formation and is made of the rRNA of the central loop and proximal nucleotides of Domain V, as well as the A-loop (loop of hairpin 92) and P-loop (loop of hairpin 80, Figure 2) [63]. The third Ψ is conserved only in eukaryotes and is located at position 2621 (2586 *E. coli *numbering) between hairpin 90 and 93 in the central loop of domain V, immediately adjacent to rRNA contacts with the P-site tRNA (terminal A of CCA at position 76) [49,63].

Three 2'-*O*-ribose methylations were also found: one G_m _is located at position 1950 (1909) in the structurally conserved helix 69 of domain IV contacting nucleotide 12 of the tRNA in the P site [49], while the conserved tandem U_m_G_m_, is located at positions 2587/2588 (2552/2553 *E. coli *numbering) in hairpin 92, also called A-loop or peptidyltransferase loop of domain V, that contacts acceptor end of tRNA in the A-site of the ribosome. In the case of *H. marismortui*, mutagenesis studies [64] and X-ray crystallography [63] demonstrated the existence of base pairing between G_m_-2588 (2553) and C-75 of CCA end of tRNA in the A-site of the ribosome, thus implicating a role in the peptidyl transfer reaction.

Lastly, two base methylations (m^1^A, a modified nucleoside bearing a positive charge and m^3^U) are present respectively at position 628 (571) in hairpin 25.1 of domain II and at position 2619 (2584) of domain V, between hairpins 90 and 93. The importance of each of these two modified nucleotides (not modified in *E. coli*) for maintenance of the archaeal ribosome architecture or translation is not known. However, since m^3^U- 2619 (2584) is in the central loop of Domain V and immediately adjacent to rRNA contacts with P-site tRNA[63], a fact revealed early on by affinity labeling results showing *E. coli *2584 adjacent the CCA end of P-site RNA [65,66], this suggest a role in peptidyltransferase activity. Moreover, the absence of the U-methylation in the homologous position in *H. salinarium *23S rRNA confers resistance to sparsomycin, and antibiotic that normally binds to the peptidyl transferase center [67].

In domain V of 23S RNA of *H. salinarium*, an additional Ψ-2606 (2580) at the bottom of helix 90, not present in rRNA of *H. marismortui*, has been reported [32]. However, as no homolog of the bacterial RluC-type, or the eukaryotic Pus5p-type of enzymes responsible for the formation of this Ψ in the LSU rRNA of respectively *E. coli *and *S. cerevisiae *can be found in the genome *H. volcanii *(see below), its presence in the 23S rRNA of *H. volcanii *is highly unlikely.

#### The case of 5S rRNA

As no modifications were detected in the 5S rRNA of the two halophilic archaea *H. halobium *and *H. marismortui *[68,69], we predicted that *H. volcanii *would also lack modifications in this rRNA. A 2'*O*-methylcytosine (Cm) at a conserved C-position (position 32) has been reported only in the 5S rRNA of the thermophiles *Sulfolobus acidocaldarius *[69] and *S. solfataricus *[68], while in the hyperthermophilic *Pyrodictium occultum *the base at the same location (position 35 in *P. occultum*) is further acetylated into ac^4^Cm. Both derivatives ac^4^C and ac^4^Cm coexist, indicating incomplete modification of C-35 under the conditions the cells were grown before extraction of the RNA [68,69]. The same is true for other modified nucleotides in the 16+23 S rRNAs.

### A complete inventory of tRNA genes (tRNomics)

The genome of *Haloferax volcanii*, strain D2 (4,012,900 nt) comprises one chromosome (2,847,757 bp) in several identical copies (up to 20 [70]) and four smaller plasmids (pHV1:85,092 bp, pHV2:6,359 bp, pHV3:437,906 bp and pHV4:635,786 bp). All tRNA genes are located on the chromosome. This mesophilic halophile exhibits the typical archaeal tRNA set [71] which is characterized by 46 distinct anticodons able to read all 61 sense codons (see details below). The extra G nucleotide at position 0 of tDNA-His (GTG) is encoded in the genome but none of the CCA 3' terminal sequence of tDNAs are present. The list of tDNA sequences in linear and cloverleaf forms is given in Additional file 2. Remarkably the sequences of each mature tRNA (as sequenced by Gupta [28,29]) and corresponding tDNAs as identified above perfectly match. The only sequences of mature tRNAs that are missing from Gupta's analysis are those specific for tRNA-Val (anticodon UAC), tRNA-Ser (UGA), tRNA-Thr (UGU), tRNA-Gln (UUG), tRNA-Arg (UCU) and tRNA-Arg (CCU), all but one harboring a T34 wobble base in the corresponding tRNA gene. As stated in the original work [28], the missing tRNAs probably correspond to minor isoacceptor species that co-migrated with one of the major species and therefore were impossible to isolate and sequence.

Six tRNA genes are present in two copies, raising the total number of tRNA genes from 46 to 52. Among these six pairs, five are perfect duplicates (from positions 1 to 73), while the two tDNA-Gly (GCC) differ by the two base pairs 4–69 and 5–68 (CG and TA versus TA and CG, respectively) as previously noted [28]. Three of these tRNA pairs are organized in direct tandems with a short distance between the two genes: 2 × tDNA-Gly (GCC), 12 nt; 2 × tDNA-Asp (GTC), 29 nt; 2 × tDNA-Val (GAC), 45 nt probably revealing a recent gene duplication. The two tDNA-Ala (TGC) are each embedded in the two copies of the ribosomal operon (between 16S and 23S rRNA genes). Other tDNAs are randomly distributed throughout the genome; the next closest distance between two tDNAs being 96 nt.

As only one gene exists for the majority of tRNAs harboring each a distinct anticodon, large differences must exist either in the expression levels of individual tDNAs, or in the half-life of individual mature tRNAs (or both). Indeed the steady state concentrations within the cell of the major tRNAs (reading most used codons) must be higher than those of minor tRNAs (reading rare codons). The regulation of the expression of the different tDNAs is yet to be elucidated in *H. volcanii *and in all other Archaea (discussed in [71]). Its is possible that tRNA stability depends on factors similar to those identified in yeast (reviewed in [72]).

Only three genes carry introns and in contrast with many other archaea (see [73]), all are found at the canonical position 37/38. The three genes, tDNA-Met (ATG) (intron of 75 nt), tDNA-Gln (CAA) (intron of 31 nt) and tDNA-Trp (intron of 103 nt), display a nearly perfect **hBHBh' **motif [73] with the so-called **h **helix being the anticodon stem and the so-called **h' **helix being 3-, 8- and 2-bp long, respectively (see [73] and Additional file 3). Pre-tRNA-Trp is unique as it contains the C/D and C'/D' boxes that allow methylation of 2' hydroxyl of the ribose at positions 34 and 39 in the intron sequence [74-77] – see also below).

As always in Archaea and Bacteria but not in Eukarya [71], three different tDNAs bearing the (CAT) anticodon are present: the initiator tDNA-Met (CAT), the elongator tDNA-Met (CAT) and the tDNA-Ile (CAT). In this last case, the final identity of the mature functional tRNA-Ile depends on post-transcriptional modification of C-34 into an as yet unknown modified C-derivative (see below).

### Codon decoding strategy

The sequences of the 46 tRNAs harboring a distinct anticodon (or tDNA when the sequence of mature tRNA is not available) are listed in Figure 3 from the wobble base at position 34 to nucleotide-39 (the proximal first base pair of the anticodon stem). This figure allows us to define the codon decoding strategy in a halophilic archaeon. It appears that: i) a systematic 'A-34 sparing' strategy is found, allowing the decoding of all pyrimidine ending codons (NN.U/C) by one tRNA harboring a G_34_. N'N' anticodon (N stating any of the 4 canonical nucleotides, N' its complementary Watson-Crick counterpart and G-34 is never post-transcriptionally modified). The presence of G-34 in tRNA-Arg (GCG) of the four codons decoding box is remarkable as the corresponding nucleoside is always an A-34 (in fact post-transcriptionally deaminated into inosine-34) in all Eukarya and most Bacteria [71]; ii) no 'C-34 sparing' strategy is used, that would require a U_34_-containing tRNA to decode a codon ending with G-3, while in the majority of Bacteria such a situation is frequent (see [71]). Thus in *H. volcanii*, the only wobbling-type case of decoding during translation of the mRNA is between a G_34_-containing tRNA and a codon ending with a U-3. An acetyl group is present on *N4 *of C-34 in many C_34_-containing tRNAs, and many of these tRNAs seem to be only partially modified [28]. The presence of ac^4^C at the wobble position of tRNAs is unique to Archaea, with the exception of the elongator tRNA-Met (ac^4^C.AU) in *E. coli *[78]. However, the same modification has been found at position 12 in the D-arm of some tRNA-Leu and tRNA-Ser molecules of *S. cerevisiae *[79] and in the 5S rRNA of some thermophilic archaea (see above). This modified nucleotide exhibits an exceptional conformational rigidity when embedded in an RNA molecule [80,81]. Its presence in the wobble position probably allows better binding of the tRNA to the cognate codon, possibly helps the tRNA to discriminate against codons ending with A [78] and to aid in phase maintenance during translation; iii) the rare isoleucine AUA codon is translated by a minor tRNA-Ile, like in all bacteria. It harbors a unique type of modified cytidine able to discriminate against the Met-AUG codon. In *E. coli*, (and all Bacteria and eukaryotic mitochondria), this C-34 residue is always modified into lysidine (k^2^C, [82], reviewed in [83]); while in Archaea, the chemical structure of the modified cytosine-34 remains to be identified ([84] see also below); iv) due to lack of sequence information about many of the mature U_34_-containing tRNAs the identification of the chemical nature of the modified U (indicated as U* in the original works of Gupta and '?U' in Figure 3) will require the discovery of potential U-34 modifying enzymes in the genome of *H. volcanii *(see below) or additional analytical experiments; v) without exception, three isoacceptor tRNAs are always used to decode four synonymous codons in the four codons decoding boxes and two isoacceptor tRNAs for decoding the two purine-ending codons (NN.G/A) in the split codon boxes. Thus altogether 45 elongator tRNA and one additional initiator tRNA-Met are required to decode the 61 sense codons in *H. volcanii*. From the early work of Bayley and Griffiths [85], it is known that accuracy of translation of synthetic homopolymers by extracts of the extreme halophilic bacterium *H. cutirubrum*, and probably all halophiles, requires the presence of very high salt concentration (up to 4 M).

### Genes coding for transfer RNA modification enzymes (Modomics)

Biochemical analysis using as substrate T7-transcripts of tRNA genes lacking all the modified nucleosides, allows enzymatic activities for producing pseudouridine and several base-methylated derivatives in tRNA, such as m^1^A-57, m^1^I-57, C_m_-56, m^1^Ψ-54, m^5^C-49 and m^2^_2_G-26 to be demonstrated in cell-free extracts of *H. volcanii *[86], but none of the corresponding genes were identified. Only recently were the genes coding for the multiprotein complex that use guide RNA to methylate the 2'-hydroxyl of cytosine-34 and uridine-39 in *H. volcanii *tRNA-Trp characterized [74,75]. In other Archaeal species (mainly in *M. jannaschii *and *P. furiosus *or *P. abyssi*), genes coding for several tRNA modification enzymes have been not only identified, but also experimentally validated. These were used to easily predict the *H. volcanii *orthologs with good confidence (Table 1). These include the enzymes that introduce the m^2^G/m^2^_2_G-10, m^1^G-37, and m^1^A-57 modifications (references are given in Table 1). A protein homologous to the key enzyme transglycosyltransferase (TGT) responsible for the insertion of the G^+ ^precursor preQ_0 _in *M. jannaschii *tRNA [87] is also found in *H. volcanii*. The genes involved the synthesis of preQ_0 _and in the conversion of preQ_0 _to G^+ ^after its insertion in tRNA, are not known in any Archaeal organisms to date. They are currently being identified in our laboratory at the University of Florida in Gainesville and will be described elsewhere. Another set of tRNA modification enzymes that introduce the Ψ-13, m^2^G/m^2^_2_G-26 and t^6^A-37 modifications respectively can be predicted by homology with yeast and/or *E. coli *experimentally validated orthologs (Table 1). For the 12 remaining modifications, the prediction process is less straightforward because the homology scores with the experimentally validated yeast or bacterial homologs are too low, paralog families complicate the analysis or the corresponding gene has not been identified in any species. These are discussed separately below.

**Table 1:** Predictions of *H. volcanii *tRNA modifications genes

**Pos.**	**Mod.**	**tRNA (see Additional file **1)	**Prediction method**	***Hv *orf**^a^	**COG**	**Comments **^b^
**10**	m^2^G/m^2^_2_G	G1-4/R1, D1, Q1, E1-2, H1, P1-3	Homology with *P. abyssi *PAB1283*[177]	HVO_0156	1041	
**13**	Ψ	R1, D1, Q1, E1-2, G1-2, H1, Me, Mi, P1-3, S1,T1,V1-2	Homology with *E. coli *TruD*[178]	HVO_0658	0585	1Z2Z
**15**	G^+^	R2-3, N1, C1, I2, L1-5, K1-2, Me, Mi, F1, P1, P3, S1-3,T1-2, W1, Y1, V2	Homology with *M. jannaschii *MJ0436*[87]	HVO_2001	0343	1IQ8
**22**	Ψ	Me	Cbf5 without Guide RNA?			See text
**26**	m^2^G m^2^_2_G	A3, R3, L4/A2,R2,I1-2, L1-3,L5,K1-2,S1-3,T1-2, W1	Homology with *P. furiosus *protein PF1871*[179]	HVO_0236	1867	2DUL
**28**	Ψ	I1	Cbf5 without Guide RNA?			See Text
**32**	Cm	K1-2,W1, Y1	Homology with *E. coli *protein TrmH*[91].	HVO_2906	0565	

**34**	Cm	Me,W1	aFib +sRNA* [75]			See Additional files 4 and 5
	*?mcm*^5^*s*^2^*U?*	R3, E2, G2, K2, L5	Homology with yeast Trm9* [180], Elp3* [125]. Tuc1* [123]	HVO_0574 + HVO_2888 + HVO_0580	2226 1243 0037	See text
	*cmo*^5^*U*	?		?	?	see text
	C*	I2	Prediction this work	HVO_0339 or HVO_0697	1571 or 2047	See text
	ac^4^C	Q1, E1, K1,P1, S1	Prediction this work	HVO_2736	1444	See text
**37**	t^6^A	N1,I1-2, K1-2, Me not Mi, S3, T1-2	Homology *M. maripaludis *protein MMP0186*[181]	HVO_0253	0009	2EQA
	m^1^G	R1-3, C1, Q1, E2, H1, L1-5, F1, P1-3, W1, Y1	Homology with *M. jannaschii *MJ0883*[182]	HVO_0929	0336	2FRN

**38-40**	Ψ_38_,Ψ_39_	38 = P1/39 = L2, L4, K1, Me, F1, S1, Y1	Homology with Yeast Pus3* [101]	HVO_1852	0101	
**39**	Um	W1	aFib + sRNA [75]			See Additional file 4
**39-40**	m^5^C	39 = L1/40 = I1	Homology with *P. abyssi *PAB1947* [110]	HVO_1594	0144	
**48,49**	m^5^C	Almost all except L4, D1, Q1, H1, Mi	Homology with *P. abyssi *PAB1947* [110]	HVO_1594	0144	
**52**	Ψ	K1	Cbf5 without sRNA?			See text
**54 and 55**	Ψ	All	Cbf5 without sRNA? and/or homology to *P. furiosus *PF1139 (PsuX) [98]	HVO_2493 HVO_1979	0103 1258	See text 2V9K
	m^1^Ψ	all except Q1,H1	Prediction [93]	HVO_1989	1901	See text 2QMM
**56**	Cm	All	Homology with *P. abyssi *PAB1040* [94]	HVO_1173	1303	2O3A
**57**	m^1^A		Homology with *P. abyssi *PAB0283* [111]	HVO_1383	2519	1YB2
	m^1^I	All harboring A57 except H1	Prediction this work	HVO_2747	1491	See text; 2I5H

^a^RefSeq annotation in archaea.ucsc.edu/; ^b^if the structure of an Archaeal member of the family is available the PDB code is given ; *Experimentally verified.

#### C_m_/U_m _residues

In *H. volcanii *tRNA, 2'-*O*-methylation of ribose occurs in four positions, 32, 34, 39 and 56 (Figure 1A). As stated above, C_m_-34 and U_m_-39 in the anticodon branch of tRNA-Trp are formed by the guide RNA machinery that includes the Fibrillarin enzyme (aFib) and accessory proteins Nop56/58 and L7Ae [88-90], all encoded in the genome of *H. volcanii *(Table 2). The RNA antisense bearing the C/D and C'/D' boxes is part of the pre-tRNA sequence and includes the long intron of 103 nt, a situation that exists also in pre-tRNA-Trp from at least 29 archaea (see Additional file 4) [76,77]. The mechanism by which the 'intronic' antisense sequence acts *in vivo in cis *to 'self' induce the 2'-*O*-methylations of C-34 and U-39 in pre-tRNA-Trp, or *in trans *by acting on an other molecule of pre-tRNA-Trp, is still an open question. However *in vitro *experiments favor a trans-acting box C/D snRNA guided mechanism [75]. In the case of intron-containing tRNA-Met, C_m_-34 is also guided by a sRNA (see Additional file 5) but here the C/D antisense RNA is not intronic but exonic as described for the C/D box sRNA sR49, which was predicted to guide the modification of Cm-34 in the tRNA-Met of *Pyrococcus *[74]. We identified 18 sRNA candidates to guide the modification of Cm-34. We found a candidate in the genome of *H. walsbyi *for which no tRNA-Met containing an intron could be identified in the genomic sequence available at NCBI. Thanks to the target region, we also identified the tRNA-Met containing the intron. Our analysis reveals that sRNA guiding formation of 2'-*O*-methyl ribose at position 34 and 39 in pre-tRNA-Trp is always intronic, while the formation of the same C_m_-34 in pre- tRNA-Met is always exonic. In both cases, part of the intron sequence is involved in base pairing with sRNA, thus 2'-*O*-methylation at ribose in position 34 and 39 in pre-tRNA-Trp and in position 34 in pre-tRNA-Met have to occur before intron splicing [77].

**Table 2:** Predictions of *H. volcanii *rRNA modifications genes

**Position**	**Modification**	**Prediction method**	***Hv *orf^a^**	**COG**	**Comments^b^**
5S					
None	None				
16S^C^					
910 (966)	acp^3^U	Prediction this work	HVO_0390	2016	See text, 1Q7H
1352 (1404)	C* = N330		?	?	See text
1432 (1500)	m^6^A	Prediction this work	HVO_1475	2263	See text, 1QAN
1450+1451 (1518+1519)	m^6^_2_A	Homology with *M. jannaschii *RsmA* [137]	HVO_2746	0030	

23S Data From *H. marismortui*^D^					
628 (571)	m^1^A	Prediction, weak homology with *E. coli *RlmA [146]	HVO_0309	2226	
1950 (1909)	Gm	aFib+ sRNA			See Additional file 8
1956+1958 (1915+1917)	Ψ,Ψ	Cbf5+ sRNA			See Additional file 8
2587 (2552)	U_m_	Homology with *E. coli *RlmE [138]	HVO_0180	1189	
2588 (2553)	G_m_	Prediction RlmE?	HVO_0180	1189	See text
2619 (2584)	m^3^U	Prediction, this work and [93]	HVO_2565	2016	See text, 1K3R
2621 (2586)	Ψ	Cbf5+ sRNA			See Additional file 8

**Guide RNA protein machinery**					
Cbf5		Homology *P. furiosus *Cbf5* [183]	HVO_2493	0103	2AUS, 2RFK, 2APO
Gar1p		Homology *P. furiosus *Gar1p* [183]	HVO_1108	3277	2HVY, 2EY4 2RFK
Nop10		Homology *P. furiosus *Nop10* [183]	HVO_0698	2260	2AUS
L7Ae		Homology *S. solfataricus *L7* [184]	HVO_2737	1358	1PXW, 2FC3, 1RLG, 1SDS, 2QA4
Fibrillarin		Homology with *S. solfataricus *aFib* [184]	HVO_1669	1889	1NT2, 1PRY 1G8S, 1FBN, 1G8A
Nop56/58		Homology *S. solfataricus *Nop56/58* [184]	HVO_1670	1498	1NT2

^a^RefSeq annotation in archaea.ucsc.edu/;^b^if the structure of an Archaeal member of the family is available the PDB code is given ; *Experimentally verified; ^c ^*H. volcanii *numbering corresponding *E. coli *numbering given in brackets; ^d ^*H. marismortui *positions corresponding *E. coli *position in brackets

Remarkably, Halobacteria show more degenerated C, C', D and D' boxes and a longer region between D' and C' boxes (19 to 21 pb) than other orders (4 to 10 pb). In contrast, the insertion of C_m_-32 found in the anticodon loop of four tRNAs (two specific for Lys, one for Tyr and one for Trp) and of C_m_-56 found in all *H. volcanii *tRNAs (with no exceptions; see Additional file 1), is almost certainly catalyzed by non RNA guided enzymes. Indeed, a solid homolog of the TrmH (YhfQ) protein that has been found to catalyze the formation of X_m_-32 in *E. coli *[91], is present in the genome of *H. volcanii *(Table 1). It is the only member of the SpoU family [92,93] found in this organism. For C_m_-56 in the Ψ-loop, a strong homolog of the *P. abyssi *protein found to catalyze this reaction *in vitro *([94] and reviewed in [95]), can be identified in the genome of *H. volcanii *(Table 1).

#### Ψ residues

Apart from Ψ-13 which is most certainly modified by the TruD ortholog (HVO_0658, belonging to COG0585, see table 1), seven other Ψ residues? are present in *H. volcanii *tRNA at positions 22, 28, 38, 39, 52, 54 and 55 (Figure 1). Ψ-55 is a universal modification inserted in yeast by Pus4p [96] and in *E. coli *by TruB [97], both belonging to the same COG0103. The only homolog of these two proteins that can be identified in the *H. volcanii *genome is Cbf5p, which is the catalytic subunit of the guide machinery (see below). However, recent work from different laboratories have shown that *in vitro*, Cbf5p can modify U-54 in tRNA, as well as in rRNA, in a guide-independent fashion, the enzymatic reaction being stimulated by the presence of Nop10p [98-100]. Psu10p from *P. furiosus*, that is not part of the TruB/Cbf5p family of proteins (COG0103) but is instead a member of the COG1258 family (Table 1), can also introduce the Ψ-55 modification in archaeal tRNAs *in vitro*. This observation has been validated by complementation experiment using an *E. coli truB *mutant [98]. It is however still not clear which of the two enzymes (Cbf5p and/or Pus10p) is responsible for the formation of Ψ-55 (as well as of Psi-54) in Archaeal tRNAs *in vivo*. As discussed in [99], the possibility exists that each of the two Ψ-55 forming enzymatic systems act on distinct sets of tRNAs. It is worthy of mention that no Psu10p homolog is found in *N. equitans*, whereas a genes coding for Cbf5p and Nop10 homologs are detected (see "Archaeal rRNA modification" subsystem in the SEED database for sequences). Unfortunately, no evidence for the presence or absence of Ψ-55 in any of the tRNAs, or of Ψ in rRNA is available for this organism.

Other quasi universal Ψ. modifications are Ψ-38/39 of the anticodon branch inserted in yeast by Pus3p [101] and in *E. coli *by TruA [102], both members of the COG0101 family. Only one protein of this family could be identified in *H. volcanii *(Table 1). Its homology with both the *E. coli *and the yeast GOG0101 members is quite low but multiple sequence alignments using clustalw [103] confirmed that the critical TruA specific active site consensus sequence (XXXRTD) [104] is conserved in the *Haloferax *protein. No homologs of yeast Pusp 1- 9 [105] or *E. coli *TruABCD (reviewed in [13]) could be identified in the *H. volcanii *genome, leaving Ψ 's at positions 22, 28 and 52 with no corresponding candidate pseudouridine synthase. The corresponding uridines are located in helical regions (at least in the fully mature tRNA), thus perhaps relatively inaccessible to the enzyme, and these are isomerized to Ψ only in one tRNA species, whereas the other Ψ modifications, at least those in positions 38, 54 and 55 are located in loops and found in several tRNAs (see Additional file 1). This led us to suppose they may be introduced at a very early stage of the tRNA biosynthesis, possibly still during transcription and when the cloverleaf structure of the nascent pre-tRNA is not yet formed, potentially by the RNA-guided machinery including Cbf5p, Nop10p, Gar1p and L7Ae homologs (Table 2). However no H/ACA snRNA could be identified in *H. volcanii*. This could be due to a high divergence of H/ACA RNA structures in *H. volcanii *or, as discussed above in the case of Ψ-55 formation, to a guide RNA independent pseudouridylation by the Cbf5p/Nop10p dependent machinery acting during transcription on an as yet unfinished tRNA molecule composed of stems and loops. One cannot however rule out that an as yet unidentified pseudouridine synthase family is present in this organism.

#### m^5^C residues

Four positions 39, 40, 48, 49 are modified to m^5^C in *H. volcanii*. In yeast, Trm4p is not site-specific and introduces this modification at several positions in tRNA molecules [106]. Members of this huge family of proteins (COG0144) are however difficult to annotate by sequence alone as some also modify rRNA [107-109]. Recently one of the five COG0144 members from *P. abyssi *(PAB1947) was found to catalyze *in vitro *the formation of m^5^C at several positions in tRNA, including positions 48 and 49 [110]. *H. volcanii *has just one member of this family (HVO_1594) that is highly similar to PAB1947, and ribosomal RNA of this organism does not contain any m^5^C (see Figures 2A–D). Hence, it is highly probable that HVO_1594 is the only RNA:m^5^C methyltransferase that modifies the four cytosines found in the sequenced tRNAs of *H. volcanii *(Figure 1). The presence of additional m^5^C residues at positions 50–52 in some tRNAs of *H. cutirubrum *probably also result from the action in this organism of a unique multi-site specific tRNA:m^5^C methyltransferase.

#### I_57 _and m^1^I_57_

**Figure 4:**
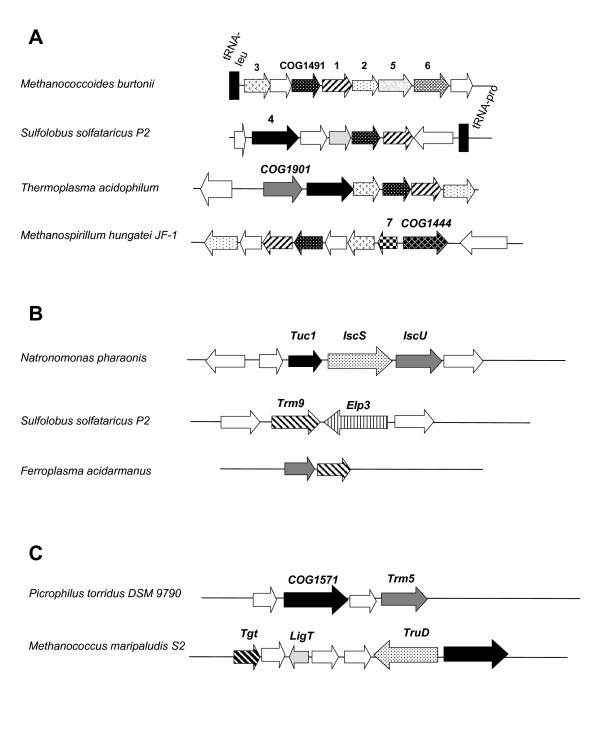
**(A) Clustering of COG1491, COG1901and COG1444 with translation gene.** 1 = KsgA [Dimethyladenosine transferase (EC 2.1.1.-)]; 2 = HemK (Methylase of polypeptide chain release factors); 3 = L21p (LSU ribosomal protein L21p); 4 = PsuX (Pus10 family see [98] and text); 5 = YhfQ (methylase potentialy involve in C_m_32 methylation of tRNA, see text); 6 = S3Ae (SSU ribosomal protein S3Ae);**7 **= Ef1b (Translation elongation factor 1 beta subunit) **(B) **Clustering of Tuc1, Elp3 and Trm9 family genes with sulfur transfer enzymes encoding genes (see text for abbreviations).**(C) **Clustering of COG1571 with RNA processing genes Tgt = tRNA-guanine transglycosylase; LigT = 2'-5' RNA ligase (EC 6.5.1.-); Trm5 = tRNA (Guanine37-N1)-methyltransferase (EC 2.1.1.31); TruD = tRNA pseudouridine 13 synthase (EC 4.2.1.-). The full analysis is available in the "Archaeal tRNA modification" and "Archaeal rRNA modification" subsystems in the SEED database.

In *H. volcanii*, the only inosine (deaminated adenosine, in the form of m^1^I) residue is found at position 57 of the majority of tRNAs (Figure 1, see also Additional file 1). Enzymatic formation of the doubly modified m^1^I occurs in two strictly sequential steps. The first step is the methylation of A-57 catalyzed by the tRNA:m^1^A methyltransferase of the *P. abyssi *TrmI family (COG2519, [111]) (Table 1). Then deamination of m^1^A-57 occurs by a tRNA:m^1^A-specific deaminase [86,112], that is different from other tRNA deaminases such as Tad1p and Tad2p/Tad3p catalyzing the formation of inosine from adenosine in position 37 and 34 respectively in *S. cerevisiae *tRNAs [113-115] or TadA catalyzing the site-specific formation of inosine-34 exclusively in tRNA-Arg (anticodon AGC)[116], as we could not identify any homologs of these families in the *H. volcanii *genome. We searched for protein families specifically conserved in all Archaea but absent in Eukarya, with RNA binding domains. One candidate is the COG1491 family. It is annotated as an RNA-binding protein as the structure of the *A. fulgidus *family member (PDB: 2I5H) showed that the N-terminal domain is similar to many nucleic acid binding protein with the presence of a characteristic S1 domain [117]. Analysis of a clustalw sequence alignment reveals a highly conserved histidine residue in a motif [R,K] [L,M]H [A,S,T,Q,M]L [E,Q,N] (Figure 4A) that is similar to the adenosine deaminase "motif I" found in all adenosine deaminases [113].

#### m^1 ^Ψ

The methylation at position 54 is a hallmark of Archaea, except in Thermococcales where m^5^U54 is found [118]. COG1901 proteins that are part of SPOUT superfamily have been predicted as candidates for this missing methylase [93] and genes of this family do indeed cluster with Psu10p in several genomes (Figure 4A). However, it is present in organisms that are expected to have m^5^U and not m^1^Ψ at this position such as the *Pyrococci *[118]. Experimental validation is required to ascertain the function of this putative Ψ-dependent methyltransferase (work in progress).

#### Modified uridine-34 derivatives

As a rule U_34_-harboring tRNAs belonging to the split codon boxes corresponding to Leu (UAA), Gln (UAG), Lys (UUU), Glu (UUC) and Arg (UCU) need to discriminate for the NN-Purine codons and not miscode for the NN-pyrimidine codons. Only certain types of modified U-34 can perform this task [119](reviewed in: [120]). In contrast, tRNAs bearing unmodified U-34 are able to decode codons ending with purines and pyrimidines, such as those found in the four codon decoding boxes as in *Mycoplasma *for example [34]. As expected a modified U! (of which the chemical identity remains to be determined) was identified in naturally occurring tRNAs of *H. volcanii *specific for Lys (U!UU) and Glu (U!UC) [28,29]. However for tRNA-Leu (?UAA) and tRNA-Arg (?UCU), the identity of U-34 is yet to be determined as these tRNAs remain to be sequenced (Figure 3 and Additional file 1). Curiously, U-34 of *H. volcanii *tRNA-Leu (UAA) was reported not to be modified, while the U-34 residue in tRNA-Leu (?UAG), tRNA-Arg (?UCG) and tRNA-Gly (?UCC) belonging to the four codons boxes appears to be (Figure 3). Unexpectedly, U-34 in tRNA-Pro (UGG) and tRNA-Ala (UGC) is not modified [28,29], while the corresponding four codons decoding boxes contains two other tRNAs (one with G-34 and the other one with C-34) able to decode the other codons, except the one ending with A (see Figure 3). Thus the pattern of modified/unmodified U-34 in *H. volcanii *tRNAs is non-canonical and the exact chemical nature of the U! in the different tRNAs of *H. volcanii *remains an enigma [as well as in the few other archaeal tRNAs sequenced so far [4].

In the case of tRNAs specific for Gln, Lys and Glu in *H. volcanii*, a thiolated U-34 derivative should exist, as for their bacterial and eukaryal counterparts (for examples see [121,122]). Indeed, in the genome of *H. volcanii*, a gene homolog to eukaryal Tuc1 belonging to COG0037 is found. In *S. cerevisiae*, this Tuc1 protein has recently been shown to be involved in the formation of s^2^U-34 in yeast cytoplasmic tRNAs [123]. Also, clustering of Tuc1 with IscS and IscU, the two proteins required for donating the thio compound (Figure 4B) strengthens the prediction that this family of proteins do participate in the formation of thiolated compounds. However, analysis of the *H. volcanii *genome suggests that ?U34 in tRNAs, as in many other archaeal tRNAs, is more complex than just a s^2^U. Indeed, homologs of the yeast Trm9p methylase and of the radical SAM enzyme Elp3 are also found in *H. volcanii *genome (Table 1). Trm9p is the yeast mcm^5^U/mcm^5^s^2^U tRNA carboxyl methyltransferases [124] and Elp3 is in yeast part of the elongation complex comprised of 6 proteins Elp1-6 that have all been shown to have a role in the formation of mcm^5^(s^2^)U [125]. *In vivo *the pleiotropic effect of mutations in the yeast Elp genes appear to be due to the absence of the modified base in tRNA [126]. However, out of the six eukaryal Elp proteins only homologs of Elp3 can be found in Archaea. This protein is part of the radical SAM family [127]. In *S. solfataricus*, the Elp3 and Trm9p encoding genes are also clustered (Figure 4B). Tuc1 is present in all sequenced genomes of Archaea, Elp3 is lacking only in *N. equitans*, and Trm9p homologs are found only in a limited subset of Archaea (see "Archaeal tRNA modification" subsystem referenced in the methods section). Taken together, the data suggest that the type of U-34 modification in *H. volcanii *tRNAs belonging to two codon split decoding boxes is similar but not identical to the mcm^5^s^2^U derivative found in eukaryal tRNAs.

Gupta proposed that mo^5^U-34 may exist in *H. volcanii *tRNAs[28]. In bacteria it has been shown that chorismic acid is a precursor to mo^5^U-34 formation through the ho^5^U intermediate, with the product of *cmoB *catalyzing the conversion of ho^5^U-34 to mo^5^U-34 [128]. CmoB protein is part of the methyltransferases type 11 family  that is difficult to annotate because it is so widespread. A distant homolog of CmoB was indeed identified (Table 2) and could be the potential mo^5^U synthase but this prediction is not very robust and absolutely requires experimental validation. The genes that are responsible for the formation of ho^5^U have not been identified in any organism.

#### ac^4^C-34

Is found only at position 34 in many *H. volcanii *tRNAs. In certain Archaea and Eukaryotes it has also been detected in ribosomal RNA (see above). The only known enzyme involved in ac^4^C formation is yeast Tan1 (YGL232W [79]) that does not have any homolog in *H. volcanii*. However it was predicted that Tan1 binds tRNA and carries the recognition determinants but must function in complex with yet unidentified acetylation enzymes [79], reviewed in [105]. One enzyme family COG1444, that contains an ATPase domain fused to an acetyltransferase domain was identified as a potential candidate. Genes of this family cluster with translation genes (Figure 4A). In yeast, the homolog (YNL132W) is essential [129], whereas the *E. coli ypfI *homolog is not [130], and has recently been shown to be responsible for ac^4^C formation in tRNA initiator in *E. coli *[131].

#### Lysidine/k^2^C_34 _homolog

Finally, like Bacteria, all Archaea but *N. equitans *have a minor tRNA-Ile (CUA) [71,84]. This requires the modification of C-34 to a C*. Otherwise this tRNA-Ile would be charged erroneously with methionine [132], reviewed in [83]. In all bacteria except in *Mycoplasma mobile *[34], a lysinyl group is inserted by the ATP-dependent TilS family of enzymes [133], but in Archaea the structure of C-34 modification is still not known, and no *tilS *gene has been found in *H. volcanii*. Potential candidates for a gene coding for an enzyme catalyzing the selective modification of C-34 in tRNA-Ile (CAU) should be found in all Archaea but *N. equitans *and should also be absent in the genomes of *E. coli *and *S. cerevisiae*. Using the OrthoMCL phylogenetic distribution search tool [134], we identified 10 protein families that conform to the above criteria. We favor two candidates in this list for missing C*-synthase genes: i) the nucleic acid binding protein-OB fold family (COG1571) that contains a potential RNA binding domain, and ii) the COG2047 family, annotated as an ATP-grasp superfamily. Both these families cluster with tRNA modification genes in several Archaea (Figure 4C) and follow the expected phylogenetic distribution (see "Archaeal tRNA modification" subsystem in SEED).

### Genes coding for ribosomal RNA modification enzymes

Very few Archaeal enzymes involved in the modification of ribosomal RNA have been experimentally characterized to date with the exception of the guide rRNA methylation and peudouridylation machinery enzymes (see for examples: [100,135,136]). However thus far little investigation has been performed in the case of halophilic organisms, including *H. volcanii*. The analysis below (Table 2, and references therein) is hence mainly based on comparative genomics predictions that await experimental validations.

#### m^6^_2_A

Among modification enzymes acting on rRNA that can be easily identified in *H. volcanii *by sequence homology is the archaeal member of the RsmA/Dim1p family. This enzyme introduces four methyl groups in the conserved tandem adenosine in hairpin 45 of the 16S RNA to form m^6^_2_A m^6^_2_A (positions 1450 and 1451; 1518 and 1519, *E. coli *numbering). The function of the *M. jannaschii *RsmA ortholog was experimentally confirmed [137]. A strong homolog of this enzyme is found in *H. volcanii *(Table 2).

#### 2'-O-methylated derivatives

Likewise, a homolog of RrmJ (or RlmE belonging to COG 1189) that catalyzes the formation of the quasi universally conserved U_m _at position 2552 of the hairpin 92 of bacterial 23S RNA [138] in *E. coli *(Um-2587 in *H. marismortui*) is also found in *H. volcanii *(Figure 2C and Table 2). In *S. cerevisiae *a site-specific Mrm2 enzyme introduces the same modification in the mitochondrial 21S (Um-2791) [139] but a guide RNA (SnR52) machinery is responsible for the equivalent cytoplasmic yeast 28S rRNA methylation (Um-2921) [140]. In this later case, Spb1p (of COG 1189 as Mrm2 and RlmE) can also catalyze the formation of Um-2921 (2552 *E. coli *numbering) even if its normal function is to catalyze the 2'-*O*-methylation of adjacent G-2922 [141]. Since the snRNP-dependent formation of U_m_-2921 occurs within the nucleus at an early step of the rRNA maturation process, and the action of Spb1 enzyme proceed later within the cytoplasm, only if U-2921 has not previously been fully modified in the nucleus, can Spb1p then complete the reaction [140]. In the case of U-2587 (2552) and/or G-2588 (2553) methylation in 23S RNA of *H. volcanii*, searches for potential guide RNA have been unsuccessful by using both pattern matching approaches and the dedicated SnoScan software, while in *P. abyssi*, a sR25 C/D box sRNA was predicted to guide the methylation of U-2669 (U-2552 in *E. coli*) [136]. Failure to detect the guide in the halophiles might be due to a divergent structure of the snRNAs in these organisms or could reflect the real absence of such guide RNAs for these particular methylation targets. The possibility exists that the halophilic RlmE homolog, identified above, is a multi-site specific enzyme and catalyzes both the formation of Um-2587 and Gm-2588. A precedent for such a situation is found in the enzymatic formation of m^1^A at both positions 57 and 58 of tRNA by the Pyrococcale TrmI enzyme [111], while the bacterial and eukaryal homologs (TrmI and Trm6p respectively) are strictly site-specific and methylate only A-58 in tRNAs [142,143]. Another possibility is that Halophiles have multiple paralog copies (from 3–6 copies, see 'Archaeal RNA subsystem") of COG3269 family that contain the RNA binding TRAM (TRM2 And MiaB, domain) [144] and one of these enzymes could be responsible for the formation of Um-2921.

We found at least one C/D box sRNA candidate to guide the 2'*O*-methylation of the ribose at position G-1950 (1909 in *E. coli*). Homologous sequences were found in 25 archaeal genomes (Additional file 7). Moreover, our results suggest that in *Pyrococcus*, the sR41 orphan C/D box sRNA  could modify the equivalent of G-1950 position.

#### Pseudouridines

No homologs of the multiple known *E. coli *pseudouridine synthases that modify rRNA (for review see [13]) could be identified in *H. volcanii*. As demonstrated for rRNA of Eukarya and some Archaea, such as *S. solfataricus, A. fulgidus *or *P. furiosus *and *P. abyssi*, Ψ residues could also be introduced by the guide RNA machinery and indeed, all the enzymes needed are presents in the *H. volcanii *genome (Table 2). In Eukaryotes, the equivalent of Ψ-1956 and Ψ-1958 are modified by the same H/ACA sRNA, respectively U19 in Human and snR191 in Yeast. In Archaea the equivalent of Ψ 1956 was proposed to be modified in Pyrococcales and *A. Fulgidus *respectively by Pf7 in *P. furiosus *and Afu4 in *A. Fulgidus *[145]. Recently a combination of *in silico *and experimental work identified seven H/ACA involved in pseudouridylation of rRNA in *P. abyssi *while a total of 17 Ψ residues were detected [100]. Some of these sRNA modify several positions in rRNA but clearly not all the 17 Ψ residues are accounted for, and for certain positions (such as Ψ-2603 of *P. abyssi *rRNA) the modification can be introduced *in vitro *by the Cbf5p/Nop10p dependent complex in the absence of any guide RNA [100]. Indeed Ψ-2016 (Ψ-1956) was introduce by the Pf7 homolog (Pab40) and the Afu-4 H/ACA sRNA *in vitro *but the modification equivalent to Ψ-1958 was not [100]. Pf7 contains three hairpin motifs, namely Pf7-stem-I, Pf7-stem-II and Pf7-stem-III, each one able to guide a modification. The *in silico *approach used in the present analysis allowed to identify two H/ACA sRNA hairpins, respectively HP1 and HP2, candidate to the modifications of Ψ-1956, Ψ-1958 and Ψ-2621. We did not find the homolog of Pf7-stemI hairpin, which is consistent with the absence of a fourth modification in *H. marismortuii*. HP2 is clearly the homolog of Pf7-stemIII and HP1 appear to be the homolog of Pf7-stemII. Remarkably in *H. volcanii *and other halobacteria, both hairpins are conserved but are separated by a long spacer whereas they are adjacent in thermococcales. HP2 would be able to guide Ψ 1956 and Ψ 1958 by forming alternative structures around the position to modify (see Additional file 8B) while the HP1 could target Ψ-2621 (see additional file 8A). Remarkably in *P. abyssi*, this last modification was not found experimentally [100] whereas Pab40 could adopt an alternative structure able to target this position (see Additional file 8A). Finally we did not find the homolog of Pf7-I and Pf7-II in the Crenarchaeote *Ignococcus hospitalis*. Certainly, the modification targeted is not present.

#### m^1^A

This methylated adenosine is located in hairpin 25.1 (position 628) of Domain II (Figure 2C). A weak homologs of RlmA that introduces a m^1^G in the *E. coli *large subunit in position 745 [146] can be identified (Table 2) and is a possible candidate for the formation of m^1^A, even if it is a different purine base. Indeed, during evolution, an enzyme able to methylated *N1 *in guanosine might have adapted to methylation of *N1 *in adenosine, exactly as an ancient *C5*-methylated enzyme has derived to become a *C5*-methyltransferase of uridine by simply changing few aminoacids in the active site in order to accommodate U instead of C [147,148].

#### m^3^U

This *N3 *methylated uridine (position 2619 in 23S RNA, 2584 in *E. coli*) is located between hairpins 92 and 93 (Figure 2C). A good candidate for the missing m^3^U inserting enzyme in *H. volcanii *is the protein belonging to COG2106 (Table 2). Indeed, analyzing the SPOUT family enzymes, Bujnicki and coll. [93] found that COG1385, exemplified by *E. coli *RsmE that introduces the m^3^U modification in 16S RNA [149], has a complementary phylogenetic distribution to the COG 2106 family found in Archaea and eukaryotes. Moreover, genes encoding COG2106 proteins are inserted in operons encoding for ribosomal proteins in phylogenetically diverse Archaea such as the *Pyrococci, Archeoglobus fulgidus *and *H. salinarium *(data not shown).

#### m^6^A

This methylated exocyclic NH_2 _of adenosine is located at position 1432 (1500 *E. coli *numbering) of 16S RNA in helix 44 in the 3' minor domain (Figure 2A). Compilation of modification data in the SSU RNA modification database [3] shows that the m^6^A modification found in the *H. volcanii *16S RNA can also be found at the same position in *S. solfataricus *and in three eukaryotes *Homo sapiens*, *Xenopus laevis *and *Rattus norvegicus*. By searching for genes that are present in these four organisms (and that are generally annotated as methylansferase) we identified the COG2263 family. Annotated as RNA methyltransferase or *N*^6^-DNA-methylase, members of this family are present in most archaea and many eukaryotes. The structure of the COG2263 member PH1948 was determined in complex with S-AdoMet [150] and revealed that this protein was a structural homolog of ErmC' (pdb :1QAN) that confers resistance to macrolides by introducing an *N*^6^-methylation at adenine 2058 (as *E. coli *numbering) of 23S rRNA [151]. We propose that the *H. volcanii *COG2263 member (Table 2) is also involved in m^6^A formation but in the 16S RNA, not the 23S RNA.

#### acp^3^U

This uridine bearing a 3-amino-3-carboxypropyl group on *N3 *of uridine is located at position 910 (966 *E. coli *numbering) of hairpin 31 in 3'major domain of 16S RNA (Figure 2A). It is modified to m^1^acp^3^Ψ. in all eukaryotes analyzed so far but is never present in small RNA subunits of bacteria that always have a non modified G (or m^2^G) in this position [3]. Using the phylogenetic pattern tool of the OrthoMCL database [134] we searched for genes that are conserved in mammals, *S. cerevisiae *and all Archaea but absent in all bacteria. A large collection of genes follow this pattern (89 altogether), most of them are ribosomal proteins and other translation related genes. One candidate stood out as a potential acp^3^U inserting enzyme, the COG2016 family. Proteins of this family are found in all sequenced Archaea and eukaryotes and contain a C-terminal PUA domain (Pseudo Uridine synthase and Archaeosine transglycosylase) that is often involved in RNA binding [152]. The yeast member of the COG2016 family, YER007C-A, has been shown to associate with ribosomes and a null mutant has clear translation defects [153].

Beside the putative genes identified above, a few other genes corresponding to as yet unidentified modified nucleotides need to be discovered, such as for the currently unidentified C*(N330) derivative located at position 1352 in hairpin 44 in the 3' minor domain SSU RNA (Figure 2A). N-330 is also found at the same position in the bacteria *Thermotoga maritima *[45]. Lastly, while the possibility is meager, one or two additional modified nucleotides might still exist in rRNAs of *H. volcanii*. Indeed, the full lengths of the 16S and 23S (1472 nt and 2922 nt respectively) of *H. volcanii *or of *H. marismortui *have not been explored, only the most critical regions where the probability was high to discover conserved or semi-conserved nucleotides have been investigated.

## Conclusion

The archaeaon *Haloferax volcanii *has the particularity of being a 'salt-loving' prokaryote that lives in the mildly hot and hypersaline environment of the Dead Sea (40- 50°C, 1.5-3M NaCl) where it was first isolated [27,154]. Life at such high salt concentrations is energetically costly. Indeed, to insure the osmotic balance between the cytosol and the high salt environment in which they thrive, halophiles have to accumulate and maintain high concentrations of solutes. These are mainly inorganic ions, such as KCl that can reach molar concentrations or Mg^2+^, but various organic osmotic solutes such as glycerol, trehalose and/or glycine betaine are also used [154,155]. As there are no visible compartments in the *Haloferax *cell [156], this lifestyle requires the adaptation of the entire intracellular enzymatic machinery, including RNA maturation and mRNA translation processes. Indeed, at high salt concentrations, the high molecular weight rRNA and the majority of proteins from non halophilic prokaryotes simply precipitate (reviewed in [157]).

Here we combine the identification of the whole set of functional tRNAs, including the presence of modified nucleosides (tRNomics), with the identification of most of the corresponding RNA modification enzymes (Modomics) in *H. volcanii*. This analysis allows to address: i) the peculiarities of the decoding strategies used by *H. volcanii *to read the 62 (61+1 initiator) sense codons of mRNAs; and ii) to emphasize the relative low number of modifications in halophilic t+rRNAs. This work is a logical continuation of a similar tRNomics analysis of fully sequenced genomes from the three kingdoms of life [71,73,158], later extended to the Modomics analysis of Mollicutes, a family of parasites that underwent a drastic reduction of their genomes during evolution [34]. On an evolution point of view, Halobacteriales like the euryarchaeon *H. volcanii*, and other distantly related organisms able to grow at salt concentrations above 100 g/L (1.7 M NaCl), such as certain Methanosarcinales (Archaea), Flavobacteria, Cyanobacteria and Proteobacteria (Bacteria) or a few Flagellated, Ciliates and Fungi (Eukarya), are all located on a relatively 'recent' branches of the small subunit rRNA based phylogenetic tree (see Figure 1 in [154]). Thus, emergence of halophilic organisms likely results from an adaptive-type of cellular evolution from a non-halophilic ancestor arising independently several times during the evolution of the three domains of life.

The detailed mechanism by which mRNAs are accurately decoded without slippage by the ribosome in an extremely halophiles is largely ignored. The only published study using cell-free system from *Halobacterium cutirubrum *shows that incorporation of radiolabeled amino-acids into polypeptides under the direction of synthetic polyribonucleotides, follows the same decoding rules found in non halophilic organisms, but that the accuracy of amino acids incorporation was dependent on the presence of various salts at high concentrations (KCl, NaCl, NH_4_Cl – [85]). This lead to the conclusion that the codon recognition processes are only secondarily dependent on ionic interactions and that the effect of salts was probably to enable all the macromolecular components to assume their correct secondary and tertiary configuration, a conclusion that is evident nowadays.

What is clear from the present work, is that the 52 (45 elongators + 1 initiator + 6 duplicants) tRNAs found in *H. volcanii *that read the 62 universally used sense codons (61+1 initiator) are typical of the Archaea that have been analyzed to date with a few minor differences discussed above ([71] and unpublished data). What is more interesting is that *H. volcanii *uses only 16 different types of modified nucleotides at 18 positions in the 46 mature tRNA isoacceptors, while both *E. coli *and *S. cerevisiae *use at least 28 different types of modified nucleotides at 20 and 35 different positions respectively [4,71]. As far as the type and position of modified nucleoside in tRNAs, the archaeon *H. volcanii *resembles Eukarya in some ways and Eubacteria in others (Table 3). The cases where an identical modification is found at the same position in the three kingdoms are rare (indicated in bold Table 3). The modifications that are archaeal specific by their chemical structure and/or their positions in tRNAs are also not numerous (underlined in Table 3). Examples include G^+^-15, ac^4^C-34, m^1^Ψ-54, Cm-56 and m^1^I-57 (Fig 1). Phylogenetic and structural analysis of the transglycosylase TGT catalyzing the incorporation of precursor of Archaeosine (G^+^) into tRNA, points to a common evolutionary origin with the present-day enzyme catalyzing the formation of queuosine at position 34 in many bacteria and higher eukaryotes [159], a typical case of divergent evolution. In contrast, the enzymatic formation of m^1^I at position 57 in archaeal tRNA involves a totally different set of enzymes than those needed to catalyze the formation of the same modification at position 37 in eukaryal tRNA-Ala [86], this time a case of convergent evolution. Our analysis has raised several questions that await experimental follow-up. Several predictions such as those for the genes involved in m^1^I or ac^4^C formation need to be validated. The nature of s^2^U-34 derivative that was predicted from the comparative genomic analysis but was not found in the initial tRNA sequencing work [28,29] has to be identified. We failed to find any gene coding for putative (multi) site-specific RNA pseudouridine synthase(s), nor for 'classical' box H/ACA guide RNAs with that catalyze the formation of pseudouridines at positions 22, 28 and 52. As point out above, it might well be that these Ψ 's are formed at very early step of the tRNA maturation (possibly during transcription) by the non RNA guide Cbf5p/Nop10P/Gar1 complex.

**Table 3:** Type and location of tRNA modifications of representative organisms belonging to the three domains of life Archaea (A), Eubacteria (B) and Eukarya (E)

**Kingdom Position**	**A**	**B**	**B**	**E**	**E (B)**
	
	***H. volcanii***	***M. capricolum*.**	***E. coli***	***S. cerevisiae***	***S. cer (mito)***
**10**	m^2^G/**m**^2^_2_**G**			m^2^G	-
12	**-**			ac^4^C	**-**
**13**	**Ψ**		**Ψ**	**Ψ**	**-**
**15**	**G+**				-
17/20	-	D	D	D	D
**22**	**Ψ**				-
26	m^2^G/m^2^_2_G			m^2^G/m^2^_2_G, Ψ	m^2^G/m^2^_2_G
28	Ψ			Ψ	Ψ
31	**-**			Ψ	Ψ
32	**-**		Ψ	Ψ	Ψ
**32**	**Cm**		**Cm**, Um	**Cm**, Ψ, m3C	**-**
**34**	**Cm, ?s**^2^**U**	**Cm, **Um, **s**^2^**U**	**Um, s**^2^**U**	**Cm,Um,Gm,s**^2^**U**	**-**
**34**	?m**c**m^5^U	cm**n**m^5^U	m**n**m^5^U	m**c**m^5^U	cm**n**m^5^U
**34**	C*, U*	k^2^C	k^2^C, cmo^5^U	cm^5^U, m^5^C	U*?
34	ac^4^C	I	ac^4^C, I	I	-?
37	-	m^6^A	m^6^A, ms^2^i^6^A	i^6^A	i^6^A
**37**	**m**^1^**G, t**^6^**A**	**m**^1^**G, t**^6^**A**	**m**^1^**G, t**^6^**A, m**^6^**t**^6^**A**	**m**^1^**G, t**^6^**A**	**m**^1^**G, t**^6^**A**
37	-			m^1^I, yW	-
**38**	**Ψ**		**Ψ**	**Ψ**	**Ψ**
**39**	**Ψ**	**Ψ**	**Ψ**	**Ψ**	**Ψ**
**39**	**m^5^C, U_m_**				-
40	m^5^C		Ψ	m^5^C	-
47	-		acp^3^U	D	-
48	m^5^C			m^5^C	-
49	m^5^C			m^5^C	-
**52**	Ψ				-
**54**	**Ψ, m^1^Ψ**		m^5^U	m^5^U	m^5^U
**55**	**Ψ**	**Ψ**	**Ψ**	**Ψ**	**Ψ**
**56**	**C_m_**				-
**57**	**m**^1^**I**				-
58	-			m^1^A	-
72	-				Ψ

For *H. volcanii *and yeast mitochondria [*S. cer(mito)*] all modifications are listed whereas for *E. coli*, *M. capricolum *and yeast cytoplasm those that are relevant for the comparison are indicated (for more details see [4]); modifications present in A,B,E are in bold; modification that appear specific to *H. volcanii *(and most certainly to Archaea) are in underlined bold and.

The type and location of modified nucleotides found in 16S rRNA of *H. volcanii *and in the 23S rRNA of the closely phylogenetically related *H. marismortui *were compared to those found in *E. coli *and *S. cerevisiae *(Table 4). There again the surprising feature in halophiles is the paucity of rRNA modifications with only 4 different modified nucleotides in 5 positions in the 16S rRNA (out of 1472 nt) and 6 in 8 positions in the 23S rRNA (out of 2922 nt). In *E. coli *there are 16 different types of modified nucleotides within 35 positions of the 16+23S rRNAs and in *S. cerevisiae *18+23+5S rRNAs contain at least 8 different modified nucleotides located at more than 100 positions [12,13]. Only a few of these modifications are found in all the three biological domains in rRNA analyzed to date from (in bold in Table 4). Without exception, they are located in critical functional domains of the RNA molecules, e.g. in the decoding center of the SSU rRNA (Figure 2B) and near the peptidyl transferase center of LSU rRNA (Fig 2D) manifesting their functional importance in various aspects of the dynamic process or mRNA translation (as discussed above in the data section). Their importance is further supported by the fact that the genes coding for the corresponding enzymes, as well as the sRNA guided modification machinery allowing the formation of these conserved t+rRNA modifications, are also remarkably conserved among the different domains of life, except for Gm-2588, m^1^A-628 and acp^3^U-910 that are present in eukaryotic rRNA and absent in bacterial rRNA (see Table 4).

**Table 4:** Type and location of rRNA modifications of representative organisms belonging to the three domains of life Archaea (A), Eubacteria (B) and Eukarya (E)

**pos**	**A**	**B**	**B**	**E**	**E (B)**
	
	***H. volcanii***	***M. capricolum*.**	***E. coli***	***S. cerevisiae***	***S. cer (mito)***
*SSU*					
910	acp^3^U-910	Enz for m^2^G	m^2^G-966	m^1^acp^3^Ψ-1189	-
	-		m^4^C_m_-1402	C_m_-1638	-
**1352**	C* = N330-1352		C-1404		-
	-		m^7^G-1407		-
**1432**	**m^6^A**		A-1500		-
**1450**	**m**^6^_2_**A**	Enz for **m**^6^_2_**A**	**m**^6^_2_**A-1518**	**m**^6^_2_**A-1780**	**-**
**1451**	**m**^6^_2_**A**	Enz for **m**^6^_2_**A**	**m**^6^_2_**A-1519**	**m**^6^_2_**A-1781**	**-**
*LSU*	*H. marismortui*				
628	m^1^A		U-571	m^1^A-645	-
**1950**	**Gm**		C-1909	A-2252	-
**1956**	Ψ	**Enz for Ψ**	**m**^3^**Ψ-1915**	**Ψ-2258**	**-**
**1958**	**Ψ**	**Enz for Ψ**	**Ψ-1917**	**Ψ-2260**	**-**
	-	Enz for G_m_	G_m_-2251	G_m_-2619	G_m_-2270
**2587**	**U**_m_	**Enz for U**_m_	**U**_m_**-2552**	**U**_m_**-2921**	**U**_m_**-2791**
	-		Ψ-2580	U-2949	Ψ-2918
2588	G_m_		G-2553	G_m_-2922	-
**2619**	**m**^3^**U**		U-2584	U-2953	-
**2621**	**Ψ**		U-2586	U-2955	-

Here, the numbering of the specific organisms is given; modifications present in A,B,E are in bold; modification that appear specific to *H. volcanii/H. marismortui *(and most certainly to Archaea) are in underlined bold; for *M. capricolum *the presence of the modification has been predicted from the presence of the corresponding gene [34] ; for *H. volcanii *and yeast mitochondria [*S. cer(mito)*] all modifications are listed whereas for *E. coli*, *M. capricolum *and yeast cytoplasm those that are relevant for the comparison are indicated, however, in few cases, is listed a given modification in *E. coli *or yeast rRNA that has no equivalent in *H. volcanii *but is present in the vicinity of a modified nucleotides found *H. volcanii*.

A clear positive correlation has been observed between the total number of ribose methylation sites, the eventual corresponding number of methylation guide sRNAs and the optimal temperature at which an organism is growing, suggesting an important role of this type of modification in RNA stabilization (reviewed in [160]). Clearly as the number of 2'- *O*-methyl ribose is exceptionally low in rRNA of halophiles, the rules guiding the faithful maturation of rRNA molecule, as well as the stabilization of their quaternary structure within the ribosome, might differ from other Archaea (psychrophiles, mesophiles and hyperthermophiles). Of note also is the absence of polyamines in extreme halophiles, as the slight amount of polyamines that can be detected actually originate from the culture medium (Oshima Tairo, personal communication). Polyamines, like Mg^2+ ^and other ions stabilize nucleic acids (reviewed in [161]) and also facilitate protein synthesis [162,163]. The 3D structure of the large 50S subunit of *H. marismortui *has been solved to 2.4 Angstrom resolution [164]. Analysis of the structure reveals a great number of monovalent and divalent ions as well as water molecules that are critical for the formation and stabilization of that rRNA structure. Hence, we propose that the presence of high concentration of salts (mono- and divalent) in the cytosol of *H. volcanii *has allowed the elimination of numerous rRNA and tRNA modifications as well as of polyamines biosynthesis, whose 'global' functions are to allow faithful maturation of pre-t+rRNAs and/or to stabilize the mature t+rRNAs and their association with other proteins (e.g. quaternary structure in the case of ribosome). If the functional replacement of many RNA modification by salts had indeed occurred, then modified nucleotides remaining in t+rRNA of halophilic organism must serve purposes other than stabilization of RNA architecture, such as decoding, accuracy of translation or other functions that cannot be functionally replaced by the electrostatic interactions provided by the surrounding salts. This hypothesis is corroborated with the fact that most, if not all of the modified nucleoside found in *H. volcanii/H. marismortui *rRNA are among the most evolutionary conserved modified nucleosides along organisms of the three biological domains (Table 4 and discussed above in data section). They are also among those we have pointed out as being the most refractory to reductive evolution in *Mycoplasma *[34]. This would suggest that the modifications remaining in *H. volcanii *tRNA are also critical for functions that cannot be replaced by salt and we are currently mutating all the corresponding genes to address the functional of these modifications *in vivo*.

This tRNomics and Modomics analysis of *H. volcanii *reinforces the necessity to integrate the knowledge of both t+rRNA sequences and modifications in order to understand the decoding properties of a given organisms. For most organisms this information can be derived only from comparative genomic analysis as sequence information of mature RNAs are lacking. However, to predict the presence or absence of modified nucleotides just from the analysis of the encoded genes is still quite dangerous and requires the type of systematic analysis performed here as a foundation in order to analyze other Archaeal genomes and understand of the function of RNA modification in Archaeal translation and its evolutionary importance.

## Methods

### tRNA genes searches in the *H. volcanii genome*

The complete genome of *H. volcanii *DS2 (April 2007 (haloVolc1) assembly) was obtained using the UCSC Archaeal Genome Browser [165]. The full set of tRNA genes (tDNAs) was first identified by searching the nucleotide sequence corresponding to all the archaeal-type conserved tRNA cloverleaf structures (for details see [71]). Verification with tRNAscan-SE[166] disclosed two more genes displaying anomalously low Cove score values. Close examination of the sequences revealed the presence of an anomalous G at position 58 (instead of the universal A58) in elongator tDNA-Met (CAT) (Cove score: 54.0); this remarkable sequence exception is confirmed by the tRNA sequencing [29]. The other exception is a G at position 8 (instead of the universal pyrimidine T8/C8) in tDNA-Thr (TGT) (Cove score: 44.6). This tRNA however was not sequenced, but one can observed in this tRNA that base 14, which is usually paired with base 8 (Watson-Crick A-T pair), is also exceptionally G instead of A suggesting a Hoogsteen G8-G14 base pair. The complete list of the 52 tDNAs of *H. volcanii *tabulated in a linear, as well as in a cloverleaf representations is given in Additional file 2. These 52 genes correspond to 46 different tRNAs (different anticodons) since 6 genes are present in two copies (the two copies of tDNA-Gly (GCC) slightly differ in the amino acid stem only). Only three genes bear introns: tRNA-Trp (CCA), tRNA-Gln (TTG) and tRNA-Met (CAT) – for details see text below.

### Compilation of mature tRNA sequences harboring modified nucleotides

The linear sequences of the 41 naturally occurring mature tRNAs of *H. volcanii*, as sequenced by Gupta [28,29] are listed in Additional file 1 (including the two variants of tRNA-Gly (GCC)). From comparison with the other fully sequenced tRNAs, the presence of many modified nucleotides in these tDNAs can however easily be inferred. Beside C* in the minor tRNA-Ile (C*AU) and U* in several U_34_-containing tRNAs, the chemical structures of all modified nucleotides are known. The probability of unknown modified nucleotides remaining in one of the six unsequenced tRNAs of *H. volcanii *is small. Analysis of the 12 additional sequences from other mesophilic halophiles, reveals the presence of m^5^C at positions 50 of tRNA-Thr (GGU), position 51 of tRNA-Val (CAC+GAC) and position 52 of tRNA-Arg (GCG), as well as of m^1^G at position 9 in tRNA-Val (CAC) and probably also in tRNA-His (GUG) and tRNA-Gln (CUG) in *H. cutirubrum*. These last modifications are not found in any of the sequenced tRNAs of *H. volcanii*.

### Mining genes coding for RNA modification enzymes

Most of the comparative genomic analysis to identify putative RNA modification genes was performed in the integrative SEED database[167] at . Results are made available in the "Archaeal tRNA modification" and the "Archaeal rRNA modification subsystem" on the publicly available server . Microbes online [168] was also used for clustering analysis and mining co-expression data. The phylogenetic pattern searches were performed using the signature search tool on the NMPDR server [169], the COG phylogenetic pattern search at NCBI ([170], ), the ortholog table tool at the MBGD database [171], the phylogenetic search query forms of OrthoMCL [134] or of the Integrated Microbial Genome (IMG) database [172]. Genome specific BLAST searches [173] were also performed at NCBI . Phylogenetic distribution of any given gene family was obtained through the IMG database [172]. Information on the presence of a given modification in RNA was essentially extracted from the RNA modification database [174], the tRNA database [4], the small rRNA modification database [3] and the 3D ribosomal modification map database [14] (for corresponding http, see above in Introduction section). Databases for rRNA and snoRNA that are involved in RNA-guided modifications are located at. [24] and . Additional information was extracted from specific articles cited throughout the text.

### Mining genes coding for C/D and H/ACA boxes RNA guide of RNA modifications

In archaea, C/D box sRNA contains four short conserved sequence motifs called the C box (RUGAUGA), D' box (CUGA), C' box (UGAUGA) and D box (CUGA), and one or two antisense elements. Each antisense element is 8-12nt long, is located immediately upstream of box D or D', and shows conserved complementarities spanning the site of modification. Each antisense element is the determinant of the site-specificity of the methylation site which is always the nucleotide of the target sequence paired to the fifth sRNA nucleotide upstream from the D(D') box (See Additional file 6, and [160]). Archaeal H/ACA sRNA are composed of one, two, or three stem-loop structure [145,175,176]. Each of these stem-loop structures can be described by two stems separated by an internal loop, a K-turn motif, and a conserved ANA (generally ACA) motif at the 3' end. The internal loop is composed of two single stranded regions which are complementary to a target region around the modified nucleotide. The target region itself encompasses two regions able to form the duplex by forming RNA-RNA interactions with the internal loop. These two regions are separated by UN, U being the uridine which will be converted into a pseudouridine (see Additional file 6

The C/D box and H/ACA box sRNAs responsible for a given set of modifications were searched by using PatScan and Darn! . In principle, the knowledge of presence of 2'-*O*-methyl derivatives as well as of Ψ in RNA is of great help to identify potential sRNAs. However, as *Halobacteria *may use non canonical type of sRNAs, the task is not simple. Despite this, for C/D box sRNA, we used a signature describing half of a C/D box sRNA containing a C (C') box motif, a short spacer, the antisense region and a D (D') box motif. The antisense region was modeled as a motif complementary to the sequence spanning four nucleotides before and after the target position. Each candidate was then extended either at its 5' or 3' end to obtain a complete sRNA sequence. In some cases, it was necessary to degenerate the signature (including one or two errors in C, C', D, D' and antisense regions) to obtain a good sRNA candidate. The same strategy was used for H/ACA sRNA. For H/ACA sRNA, the initial signature contained the characteristics of a stem-loop structure with the stem down to the pocket, the two 3–5 nt antisense elements surrounding the residue to modify, a K-turn (K-loop) motif and an ANA motif situated 13–16 nucleotides from the Ψ residue. For each candidate found, we searched for homologous sequences by combining pattern matching approaches and similariy searches (using NCBI-Blast against complete genomes of archaea at . Only candidates found in inter-coding sequences and showing strong homology evidence were kept as good candidates.

Comparison with known sRNA was performed by using data from the literature and available databases , ,.  and ).

### Nomenclature

All tRNA genes and mature tRNA (with their anticodon in brackets) are designated as this example: tDNA-Ile (GAT) and tRNA-Ile (GAU) respectively. The conventional numbering system for tRNA positions and the symbols used for the modified nucleosides are those adopted in the tRNA database [4]. The number after a ribonucleotide (symbolized by A, U, G, C) or its modified counterpart corresponds to its position in the tRNA molecules. In the case of rRNA, unless otherwise specified, numbers correspond to the equivalent position in the *E. coli *rRNA. Only nucleoside C* is unconventional. As discussed below, C* found at the wobble position 34 of *H. volcanii *tRNA-Ile (anticodon C*AU) corresponds to a yet incompletely characterized, probably 'lysidine-type' cytosine, while at position 1342 (1404 *E. coli *numbering) of *H. volcanii *16S rRNA, C* corresponds to another uncharacterized C-derivative of a molecular mass of 330.117 Da (N-330). Detailed chemical structures, scientific and common names corresponding to each indicated modified nucleoside and as well as of the corresponding RNA modification enzymes can be found at  and at 

## Abbreviations

COG: Cluster of Orthologous Group; ORF: open reading frame; SAM or S- AdoMet: S-Adenosyl-L-Methionine; SSU: small subunit; LSU: large subunit; PTC: peptidyl transferase center; NCBI: National Center for Biotechnology Information; RNP: ribonucleprotein; Nt: nucleotide.

## Authors' contributions

HG and VdC-L designed the study and coordinated the analysis. HG carried out the analysis of the tRNA sequences, CM searched and analyzed the tDNA sequences, CG searched and analyzed the sRNA sequences, WD did the analysis of the rRNA modification in the context of the Ribosome structure, VdC-L predicted all the modifications genes and drafted the manuscript. All authors read and approved the final manuscript.

## Supplementary Material

Additional File 1 **Sequences of the 41 mature tRNAs + 6 tDNA covering the whole decoding set of *Haloferax volcanii*.** Click here for file

Additional File 2 **Sequences of the 52 tDNAs of *Haloferax volcanii *and Genetic Code coverage.** Click here for file

Additional File 3 **The hBHBh' structure of the introns in pre-tRNA-Met (CAU), pre-tRNA-Gln (UUG) and pre-tRNA-Trp (CCA) in *Haloferax volcanii*.** Click here for file

Additional File 4 **sRNAs of *Haloferax volcanii *predicted to modify Cm34 and Um39 in tRNA-Trp.** Click here for file

Additional File 5 **sRNA of *Haloferax volcanii *predicted to modify Cm34 in tRNA-Met.** Click here for file

Additional File 7 **sRNA of *Haloferax volcanii *predicted to modify Gm1934 (Gm1950) in 23S rRNA.** Click here for file

Additional File 8 **H/ACA sRNA sequences of *Haloferax volcanii *and some homologous sequences.** Click here for file

Additional File 6 **Representation of C/D box and H/ACA sRNA in archaea.** Click here for file

Additional File 9 **Legends for Additional files** Click here for file
