# Supplementary material for: RNomics and Modomics in the halophilic archaea Haloferax volcanii: identification of RNA modification genes
Source: BMC Genomics. 2008 Oct 9;9:470. doi: 10.1186/1471-2164-9-470 (PMC2584109; doi:10.1186/1471-2164-9-470)
Supplement: Additional File 1 — Sequences of the 41 mature tRNAs + 6 tDNA covering the whole decoding set of Haloferax volcanii. [file 1471-2164-9-470-S1.doc]

**Transfer RNA population from *Halobacterium volcanii***

________________________________________________________________________________________________________

| accept| |D-domain ||anticodon domain ||variable region ||T-domain ||accept|

| stem | | D-loop || ||| extra loop | || || stem |

|0123456789**1**1111**1**1111122222222**2**2223333**3**33**3**33444444eeeeeeeeeeeeeeeeeee44**44**5555**5555**5566666666667777777

| **0**1234**5**6778900012345**6**7890123456**7**89012345111111112345222222267**89**0123**4567**8901234567890123456

| a ab 1234567 7654321

______________________________________________________________________________________________________

A1 GGC -GGGCUCGUAGAUCAGG--GGU--AGAUCACUCCCUUGGCAUGGGAGAG-------------------GC**??**CGGG**]PBO**AAUCCCGGCGAGUCCACCA

A2 CGC -GGGCUCGUAGAUCAGU--GGC--AGAUC**R**CUUCCUUCGCAAGGAAGAG-------------------GC**??**GGGG**]PBO**AAUCCCCGCGAGUCCACCA

A3 UGC -GGGCCCAUAGCUCAGU--GGU--AGAGU**L**CCUCCUUUGCAAGGAGGAU-------------------GC**??**AGGG**]PB**GAAUCCCUGUGGGUCCACCA

R1 GCG -GUCCUGAUA**R**GG**P**AGU--GGACUAUCCUCCUGGCUUGCG**K**AGCCAGGG-------------------A-C**?**GGAG**]PBO**AAUCUCCGUCAGGACGCCA

R2 CCG -GGGCCCGUAGCUCA**;**U--GGAC-AGAGU**R**CUUGGUUCCG**K**ACCAAGAU-------------------GC**?**GCGGG**]PBO**AAUCCCGUCGGGUCCGCCA

R3 UCG -GGGCGCUUAGCUCA**;**UCUGGAC-AGAGU**L**CUUGGCU**!**CG**K**ACCAAGUU-------------------GC**?**ACGGG**]PBO**AAUCCUGUAGCGCCCACCA

*R4 UCU -GgGCGCGTAGCTCAGTC-GGAC-AGAGCGTCGGACTTCTAATCCGATG-------------------GTCgCGGGTTCGAATCCCGtCGCGCtCG*

*R5 CCU -GGGCGTGTGGCCtAGT--GGAC-AgGGCGAGAGGTTCCTAACCTCTCG-------------------ATCgCGGGTTCGAATCCCGtCACGCCCG*

N1 GUU -GCCGCCGUAGCUCA**;**UU-GGU--AGAGCACCUCGCUGUU**6**ACGAGGUU-------------------GU**??**CAGG**]PB**GAGUCCUGGCGGUGGCGCCA

D1 GUC -GCCCGGGUG**R**UG**P**AGU--GGCCCAUCAUACGACCCUGUCACGGUCGUG-------------------A-CGCGGG**]PBO**AAUCCCGCCUCGGGCGCCA

C1 GCA -GCCAAGGUGGCAGA**;**UUCGGCCCAACGCAUCCGCCUGCA**K**AGCGGAAC------------------CCC**?**GCCGG**]PBO**AAUCCGGCCCUUGGCUCCA

Q1 CUG -AGUCCCAUG**R**GG**P**AGU--GGCCAAUCCUGUUGCCUU**M**UG**K**GGGCAACG-------------------A-CCCAGG**PPB**GAAUCCUGGUGGGACUACCA

## Q2 UUG -AGTCCCGTGgTGtAGC--GGCCAAtCAtAGCGGCCTTTGGAGCCGCTG-------------------A-CGGCGGTTCGAATCCGCCCGGGACTA

E1 CUC -GCUCUGUUG**R**UG**P**AGUCCGGCCAAUCAUAUCACCCU**M**UCACGGUGAUG-------------------A-C**?**AGGG**]PB**GAAUCCCUGACGGAGCACCA

E2 UUC –GCUCGGUUG**R**UG**P**AGUCCGGCCAAUCAUCUUGGCCU**!**UC**K**AGCCGAGG-------------------A-C**?**AGGG**]PBO**AAUCCCUGACCGAGCACCA

G1**a**GCC -GCG**CU**GGUA**L**UG**P**AGU--GGU--AUCACGUGACCUUGCCAUGGUCACA-------------------A-C**?**UGGG**]PBO**AAUCCCAGCC**AG**CGCACCA

G1**b**GCC -GCG**UC**GGUA**L**UG**P**AGU--GGU--AUCACGUGACCUUGCCAUGGUCACA-------------------A-C**?**UGGG**]PBO**AAUCCCAGCC**GA**CGCACCA

G2 UCC –GCACCGGUG**L**UCUAAU--GGU--AAGACAUUGGCCU**!**CCAAGCCAAUU-------------------A-U**?**UGGG**]PB**GAUUCCCAGCCGGUGCACCA

G3 CCC -GCGCCGAUG**L**UCCAGU--GGU--AGGACACGAGCUUCCCAAGCUCGGA-------------------G-C**?**CGGG**]PBO**AUUCCCGGUCGGCGCACCA

H1 GUG **G**UCCGGGUUG**R**GG**P**AGU--GGACUAUCCUUCAGCCUUGUG**K**AGGCUGAG-------------------A-CGCGGG**PPB**AAUUCUCGCACCUGGACCCA

I1 GAU -GGGCCAAUAGCUCAGUCAGGUU--GAGC**R**C**P**CGGCUGAU**6**AC**?**GGGAG-------------------GCC**?**GCGG**]PBO**AAUCCGCGUUGGCCCACCA

**I2 C**AU -GGGCCCCUAGCUCA**;**UCUGGUC-AGAGCRCUCGGCU**N**AU**6**ACCGGGUG-------------------GU**?**AUGGG**]PB**GAACCCCAUGGGGCCCACCA

L1 GAG -GCGUGGGUAGCCAA**;**CCAGGCCAACGGC**R**CAGCGUUGAG**K**G**?**GCUGUCCU-----GUAG-----AGGUC**?**GCCGG**]PBO**AAUCCGGUCCCACGCACCA

L2 CAA -GCGAGGGUAGCUAA**;**UCAGGAA-AAAGC**R**GCGGACUCAA**K**A**P**CCGCUCCC-----GUAG-----GGGUC**?**GUGGG**]PBO**AAUCCCUCCCCUCGCACCA

L3 CAG -GCAGGGAUAGCCAA**;**UCUGGCCAACGGC**R**CAGCGUUCAG**K**GCGCUGUCUC-----AUAG-----GAGUC**?**GCAGG**]PBO**AAUCCUGCUCCCUGCACCA

L4 UAA -GCGGGGGUGGCUGA**;**CCAGGCCAAAAGC**L**GCGGACUUAA**K**A**P**CCGCUCCC-----GUAG-----GGGUUCGCGAG**]PB**GAAUCUCGUCCCCCGCACCA

L5 UAG -GCGCGGGUAGCCAA**;**U--GGCCAAAGGC**R**CAGCGCU**!**AG**K**ACGCUGUGGU-----GUAG-----ACCUU**?**GCAGG**]PB**GAACCCUGUCCCGCGCACCA

K1 CUU -GGGCCGGUAGCUCA**;**UUAGGC--AGAGC**R**UCUGA**B**U**M**UU**6**A**P**CAGACG-------------------GU**?**GCG**P**G**]PBO**AAUCGCGUCCGGCCCACCA

K2 UUU -GGGCUGGUAGCUCA**;**UUAGGC--AGAGC**R**UCUGG**B**U**!**UU**6**ACCAGACG-------------------GU**?**GGGGG**]PBO**AGUCCCUCCCAGCCCGCCA

M**e** CAU -GCCCGGGUGGCU**P**A**;**CU-GGAC-A**P**AGCGCCGCACU**B**AU**6**A**P**GCGGAG-------------------AU**?**GUGGG**]PB**GGAGCCCACCCCGGGCACCA

M**i** CAU -AGCGGGAUGGGA**P**A**;**CCAGGAG-AUUCCGCCGGGCUCAUAACCCGGAG-------------------AUCGGUAG**]PBO**AAUCUACCUCCCGCUACCA

F1 GAA -GCCGCCUUAGCUCA**;**ACUGGG--AGAGCACUCGACUGAA**K**A**P**CGAGCU-------------------GU**?**CCCGG**]PBO**AAUCCGGGAGGCGGCACCA

P1 CGG -GGGCCGGUG**R**GG**P**A**;**CUUGGU--AUCCUUCGGCCUU**M**GG**KP**GGCCGUA-------------------A-**??**UCAG**]PB**GAAUCUGAGCCGGCCCACCA

P2 GGG -GGGACCGUG**R**GG**P**AGU--GGU--AUCCUCUGCCGAUGGG**K**UCGGUAGG-------------------A-C**?**UGAG**]PB**GACUCUCAGCGGUCCCACCA

P3 UGG -GGGACCGUG**R**GU**P**A**;**CCUGGU--AUACUUCGGGCCUUGG**K**UGCCCGUG-------------------A-**??**CCGG**]PBO**AAUCCGGGCGGUCCCACCA

S1 CGA -GCCGAGGUAGCC**P**A**;**CCCGGCC-AAGGC**R**GUAGAUU**M**GAAA**P**CUACU-GUCC---AUUC----GGACA-**?**GUGAG**]PBO**AAUCUCACCCUCGGCGCCA

S2 GGA -GCCAGGAUGGCCGA**;**C--GGU--AAGGC**R**CACGCCUGGAAAGCGUGU-UCCC---UCU-----GGGAU-**?**GGGGG**]PBO**AAUCCCUCUCCUGGCGCCA

S3 GCU -GUUGCGGUAGCCAA**;**CCUGGCCCAAGGC**R**CUGGGUUGCU**6**ACUCAGU-GGC----GUCAA----GCCC-**??**GGGG**]PB**GAAUCCCCGCCGCAACGCCA

# S4 TGA -GGTGGGATGGCGgAGT--GGCCTAaCGCGCCTGCCTTGAAAGCAGGT-TTcc---tcac----ggaat-CCTGGGTTCAAATCCCAGTCCCACCG

T1 GGU -GCCUGGGUAGCUPA**;**C--GGU--AAAGC**R**CGUCCUUGGU**6**AGGACGAG-------------------AC**??**CGGG**]PBO**AAUCCCGGCCUAGGCUCCA

T2 CGU -GCCGGUGUAGCUCA**;**UU-GGC--AGAGC**R**AUUCCUUCGU**6**AGGAAUAG-------------------GC**?**GAGGG**]PBO**AAUCCCUCCACCGGCUCCA

*T3 TGT -GCCAGGAGAGCATGGGC-GGTT-CATGCACTCGACTTGTAATCGAGAC-------------------TTCGTGGGTTCAAATCCCACTCCTGGCT*

W1 CCA -GGGGCUGUGGCCAA**;**CCCGGC--AUGGC**R**ACUGA**B**U**B**CA**K**A**J**CAGUCG-------------------AU**?**GGGGG**]PBO**AAUCCCUCCGGCCCCACCA

Y1 GUA -CCGCUCUUAGCUCA**;**CCUGGC--AGAGCAGCCGA**B**UGUA**K**A**P**CGGCUU-------------------GU**?**CCCCG**]PBO**AAUCGGGGAGAGCGGACCA

V1 GAC -GGGUUGGUGGUC**P**AGUCUGGUU-AUGACACCUCCUUGACAUGGAGGAG-------------------GC**?**GGCAG**]PBO**AAUCUGCCCCAACCCACCA

V2 CAC -GGGUUGGUGGUC**P**A**;**CCAGGUU-AUGACGGCUCCUUCACACGGAGCAG-------------------GC?GGCGG**]PB**GAAUCCGCCCCAACCCACCA

# V3 TAC -GGGCTCGTGGTCTAGTT-GGTT-ATGACGCGGCCTTTACAAGGCTGAG-------------------GTCGGTGGTTCGAATCCGCCCGAGCCCA

______________________________________________________________________________________________________

0123456789**1**1111**1**1111122222222**2**2223333**3**33**3**33444444eeeeeeeeeeeeeeeeeee44**44**5555**5555**5566666666667777777

**0**1234**5**6778900012345**6**7890123**4**56**7**89012345111111112345222222267**89**0123**4567**8901234567890123456

| a ab 1234567 7654321

________________________________________________________________________________________________

**Conventional one letter code for modified nucleotides in fully mature tRNAs**

Sprinzl Commun number of

code symbol occurrence Name

6 t^6^A 9 N-((9-ribofuranosylpurin-6-yl)carbamoyl)-threonine

O m^1^I 27 1-Methylinosine

B Cm 47 2'-*O*-Methylcytidine

M ac^4^C 5 N4-Acetylcytidine

? m^5^C 46 5-Methylcytidine

; G^+^ 24 Archaeosine

K m^1^G 18 1-Methylguanosine

L m^2^G 7 N2-Methylguanosine

R m^2^_2_G 25 N2,N2-Dimethylguanosine

**N** ? 1 Unknown modified Cytidine(structure not yet known by May 2008)

J Um 1 2'-*O*-methyluridine

**!** ?(s^2^)U?  5 Unknown modified Uridine (eventually 2-thiolated - see text)

P Ψ 71 Pseudouridine

] m^1^Ψ 38 1-methylpseudouridine
