# Supplementary material for: RNomics and Modomics in the halophilic archaea Haloferax volcanii: identification of RNA modification genes
Source: BMC Genomics. 2008 Oct 9;9:470. doi: 10.1186/1471-2164-9-470 (PMC2584109; doi:10.1186/1471-2164-9-470)
Supplement: Additional File 2 — Sequences of the 52 tDNAs of Haloferax volcanii and Genetic Code coverage. [file 1471-2164-9-470-S2.doc]

**Sequences of the 52 tDNAs of *Haloferax volcanii* and Genetic Code coverage**

**A/** The 52 tDNAs are listed according to 1-letter amino acid code under linear and cloverleaf forms. Under the “CAA” heading are listed the 3 nt following nt 73 in the genome. Color code used is: blue, acceptor stem; red, D-stem; cyan, anticodon stem; green, T-stem. Lower case letters are used for mismatches or GT pairing (GU in tRNA). Six tDNAS are present in two copies, note the slight difference in the acceptor stem of the two tDNA-Gly (GCC).

**B/** Genetic code coverage (tDNA Usage). Values indicate the number of genes coding for tRNA bearing the anticodon indicated.

A

base #47 or (V-arm length -4)

direction base #44 |

|chromosome base #1 optional bases intron length | | base #73 anticodon

|| start end | A box || || anticodon | | | B box | CCA AA | codon

--------- --------- _______ .. .... ...**...**. ____ _ _____ __ ... _ --- _ _____ ___ - _ ____. ....... .____ _______ _ ___ ------ ---- ---

> 1048559 1048630 GGgCTCG TA GATC AGG--GGT--A GATC A CTCCC TT GGC A - T GGGAG AGG C C CCGGG TTCAAAT CCCGG CGAGtCC A TTT **A** Ala (GGC) GCC

1

T T T 76 3'

A

tDNA **A** (GGC) GCC 5' 1 G + C

G + C

g • t 70

C + G

T + A 60

C + G |

16 G + C T A

| 10 T G G C C C A

17 - G G A | A + + + + + A

17a - C T A G C C G G G C

G + + + + 48 C T T

G G A T C

T - - A A A C 47

| | | C + G G G

20 T + A

20a C + G

20b 30 C + G 40

C + G

T T

T A

G G C

> 1599762 1599833 GGgCCCA TA gCTC AGT--GGT--A GAGt G CCTCC TT TGC A* - A GGAGG ATG C C CAGGG TTCGAAT CCCTG TGGGtCC A TGG **A** Ala (TGC) GCA

< 2770065 2769994 GGgCCCA TA gCTC AGT--GGT--A GAGt G CCTCC TT TGC A* - A GGAGG ATG C C CAGGG TTCGAAT CCCTG TGGGtCC A TGG **A** Ala (TGC) GCA

2

T G G 76 3'

A

tDNA **A** (TGC) GCA 5' 1 G + C

G + C

g • t 70

C + G

C + G 60

C + G |

16 A + T T A

| 10 T G T C C C A

17 - T G A | A + + + + + G

17a - C T C g C A G G G C

G + + + • 48 C T T

G G A G t

T - - A G A C 47

| | | C + G T G

20 C + G

20a T + A

20b 30 C + G 40

C + G

T A

T A

T G C

> 329507 329578 GGgCTCG TA GATC AGT--GGC--A GATC G CTTCC TT CGC A - A GGAAG AGG C C CGGGG TTCAAAT CCCCG CGAGtCC A TCA **A** Ala (CGC) GCG

1

T C A 76 3'

A

tDNA **A** (CGC) GCG 5' 1 G + C

G + C

g • t 70

C + G

T + A 60

C + G |

16 G + C T A

| 10 T G C C C C A

17 - T G A | A + + + + + A

17a - C T A G C G G G G C

G + + + + 48 C T T

G G A T C

C - - A G A C 47

| | | C + G G G

20 T + A

20a T + A

20b 30 C + G 40

C + G

T A

T A

C G C

> 1603383 1603459 GCCAAGG TG GCag AGTTCGGCCCA acGC A TCCGC CT GCA G - A GCGGA ACC 2 C GCCGG TTCAAAT CCGGC CCTTGGC T CTC **C** Cys (GCA) TGC

1

C T C 76 3'

T

tDNA **C** (GCA) TGC 5' 1 G + C

C + G

C + G 70

A + T

A + T 60

G + C |

16 G + C T A

| 10 T C G G C C A

17 T T G A | G + + + + + A

17a C g a C G G C C G G C

G • • + + 48 C T T

G a c G C

C C C A A A CC 2 extra arm

| | | T + A C C

20 C + G

20a C + G

20b 30 G + C 40

C + G

C A

T G

G C A

< 311725 311653 GCCCGgG TG gTGt AGT--GGCCCA tCAt A CGACC CT GTC A - C GGTCG TGA - C GCGGG TTCAAAT CCCGC CtCGGGC G TAC **D** Asp (GTC) GAC

< 311827 311755 GCCCGgG TG gTGt AGT--GGCCCA tCAt A CGACC CT GTC A - C GGTCG TGA - C GCGGG TTCAAAT CCCGC CtCGGGC G CTC **D** Asp (GTC) GAC

2

C T C 76 3'

G

tDNA **D** (GTC) GAC 5' 1 G + C

C + G

C + G 70

C + G

G + C 60

g • t |

16 G + C T A

| 10 T C G C C C A

17 - T G A | G + + + + + A

17a - t G T g G C G G G C

G • + + • 48 C T T

G t C A t

C C C A A T - 47

| | | C + G G A

20 G + C

20a A + T

20b 30 C + G 40

C + G

C C

T A

G T C

> 2646860 2646934 GCTCGGT TG gTGt AGTCCGGCCAA tCAt C TtGGC CT TTC G - A GCCgA GGA - C CAGGG TTCAAAT CCCTG ACCGAGC A TCT **E** Glu (TTC) GAA

1

T C T 76 3'

A

tDNA **E** (TTC) GAA 5' 1 G + C

C + G

T + A 70

C + G

G + C 60

G + C |

16 T + A T A

| 10 T G T C C C A

17 C T G A | G + + + + + A

17a C t G T g C A G G G C

G • + + • 48 C T T

G t C A t

C C A A C G - 47

| | | T + A G A

20 t • g

20a G + C

20b 30 G + C 40

C + G

C A

T G

T T C

> 2759237 2759311 GCTCtGT TG gTGt AGTCCGGCCAA tCAt A TCACC CT CTC A - C GGTGA TGA - C CAGGG TTCGAAT CCCTG ACgGAGC A CTT **E** Glu (CTC) GAG

1

C T T 76 3'

A

tDNA **E** (CTC) GAG 5' 1 G + C

C + G

T + A 70

C + G

t • g 60

G + C |

16 T + A T A

| 10 T G T C C C A

17 C T G A | G + + + + + G

17a C t G T g C A G G G C

G • + + • 48 C T T

G t C A t

C C A A A T - 47

| | | T + A G A

20 C + G

20a A + T

20b 30 C + G 40

C + G

C C

T A

C T C

< 439312 439239 GCCGCCT TA GCTC AGACTGGG--A GAGC A CTCGA CT GAA G - A TCGAG CTG T C CCCGG TTCAAAT CCGGG AGGCGGC A TCC **F** Phe (GAA) TTC

< 441309 441236 GCCGCCT TA GCTC AGACTGGG--A GAGC A CTCGA CT GAA G - A TCGAG CTG T C CCCGG TTCAAAT CCGGG AGGCGGC A TAT **F** Phe (GAA) TTC

2

T A T 76 3'

A

tDNA **F** (GAA) TTC 5' 1 G + C

C + G

C + G 70

G + C

C + G 60

C + G |

16 T + A T A

| 10 T G G G C C A

17 C A G A | A + + + + + A

17a T C T C G C C C G G C

G + + + + 48 C T T

G G A G C

G - - A A C T 47

| | | C + G T G

20 T + A

20a C + G

20b 30 G + C 40

A + T

C A

T G

G A A

> 2420585 2420655 GCGCTGG TA GTGt AGT--GGT--A tCAC G TGACC TT GCC A - T GGTCA CAA - C CTGGG TTCAAAT CCCAG CCAGCGC A CTT **G** Gly (GCC) GGC

1

C T T 76 3'

A

tDNA **G** (GCC) GGC 5' 1 G + C

C + G

G + C 70

C + G

T + A 60

G + C |

16 G + C T A

| 10 T G A C C C A

17 - T G A | A + + + + + A

17a - t G T G C T G G G C

G • + + + 48 C T T

G t C A C

T - - A G C - 47

| | | T + A A A

20 G + C

20a A + T

20b 30 C + G 40

C + G

T T

T A

G C C

> 2420668 2420738 GCGTCGG TA GTGt AGT--GGT--A tCAC G TGACC TT GCC A - T GGTCA CAA - C CTGGG TTCAAAT CCCAG CCGACGC A CTT **G** Gly (GCC) GGC

1

C T T 76 3'

A

tDNA **G** (GCC) GGC 5' 1 G + C

C + G

G + C 70

T + A

C + G 60

G + C |

16 G + C T A

| 10 T G A C C C A

17 - T G A | A + + + + + A

17a - t G T G C T G G G C

G • + + + 48 C T T

G t C A C

T - - A G C - 47

| | | T + A A A

20 G + C

20a A + T

20b 30 C + G 40

C + G

T T

T A

G C C

< 937934 937864 GCACCGG TG GTCT AAT--GGT--A AGAC A TTGGC CT TCC A - A GCCAA TTA - T CTGGG TTCGATT CCCAG CCGGTGC A TCT **G** Gly (TCC) GGA

1

T C T 76 3'

A

tDNA **G** (TCC) GGA 5' 1 G + C

C + G

A + T 70

C + G

C + G 60

G + C |

16 G + C T T

| 10 T G A C C C A

17 - T A A | G + + + + + G

17a - T C T G C T G G G C

G + + + + 48 T T T

G A G A C

T - - A A T - 47

| | | T + A T A

20 T + A

20a G + C

20b 30 G + C 40

C + G

C A

T A

T C C

< 1733386 1733316 GCGCCGA TG GTCC AGT--GGT--A GGAC A CGAGC TT CCC A - A GCTCG GAG - C CCGGG TTCAATT CCCGG TCGGCGC A TCT **G** Gly (CCC) GGG

1

T C T 76 3'

A

tDNA **G** (CCC) GGG 5' 1 G + C

C + G

G + C 70

C + G

C + G 60

G + C |

16 A + T T T

| 10 T G G C C C A

17 - T G A | G + + + + + A

17a - C C T G C C G G G C

G + + + + 48 C T T

G G G A C

T - - A A G - 47

| | | C + G A G

20 G + C

20a A + T

20b 30 G + C 40

C + G

T A

T A

C C C

< 259370 259298GTCCgGGT TG gGGt AGT--GGACTA tCCt T CAGCC TT GTG G - A GGCTG AGA - C GCGgG TTCAATT CtCGC ACCtGGA C CTC **H** His (GTG) CAC

1

C T C 76 3'

G + C

tDNA **H** (GTG) CAC 5' 1 T + A

C + G

C + G 70

g • t

G + C 60

G + C |

16 T + A T T

| 10 T C G C t C A

17 - T G A | G + + + • + A

17a - t G G g G C G g G C

G • + + • 48 C T T

G t C C t

A C T A T A - 47

| | | C + G G A

20 A + T

20a G + C

20b 30 C + G 40

C + G

T A

T G

G T G

> 2217987 2218060 GGGCCAA TA GCTC AGTCAGGT--T GAGC G CtCGG CT GAT A - A CCGgG AGG C C CGCGG TTCAAAT CCGCG TTGGCCC A CTC **I** Ile (GAT) ATC

1

C T C 76 3'

A

tDNA **I** (GAT) ATC 5' 1 G + C

G + C

G + C 70

C + G

C + G 60

A + T |

16 A + T T A

| 10 T G C G C C A

17 C T G A | A + + + + + A

17a A C T C G C G C G G C

G + + + + 48 C T T

G G A G C

T - - T G A C 47

| | | C + G G G

20 t • g

20a C + G

20b 30 G + C 40

G + C

C A

T A

G A T

> 1706125 1706198 GGGCTGG TA GCTC AGTTAGGC--A GAGC G TCTGG CT TTT A - A CCAGA CGG T C GgGGG TTCAAGT CCCtC CCAGCCC G TTT **K** Lys (TTT) AAA

1

T T T 76 3'

G

tDNA **K** (TTT) AAA 5' 1 G + C

G + C

G + C 70

C + G

T + A 60

G + C |

16 G + C T G

| 10 T C t C C C A

17 T T G A | A + • + + + A

17a A C T C G G g G G G C

G + + + + 48 C T T

G G A G C

C - - A G C T 47

| | | T + A G G

20 C + G

20a T + A

20b 30 G + C 40

G + C

C A

T A

T T T

< 1546223 1546150 GGGCCGG TA GCTC AGTTAGGC--A GAGC G TCTGA CT CTT A - A TCAGA CGG T C gCGtG TTCAAAT CgCGt CCGGCCC A TTC **K** Lys (CTT) AAG

1

T T C 76 3'

A

tDNA **K** (CTT) AAG 5' 1 G + C

G + C

G + C 70

C + G

C + G 60

G + C |

16 G + C T A

| 10 T t G C g C A

17 T T G A | A • + + • + A

17a A C T C G g C G t G C

G + + + + 48 C T T

G G A G C

C - - A G C T 47

| | | T + A G G

20 C + G

20a T + A

20b 30 G + C 40

A + T

C A

T A

C T T

< 2082124 2082040 GCGGGGG TG GCTg AGCCAGGCCAA aAGC G GCGGA CT TAA G - A TCCGC TCC 10 C gCGAG TTCGAAT CTCGt CCCCCGC A TTT **L** Leu (TAA) TTA

1

T T T 76 3'

A

tDNA **L** (TAA) TTA 5' 1 G + C

C + G

G + C 70

G + C

G + C 60

G + C |

16 G + C T A

| 10 T t G C T C A

17 C C G A | G • + + + + G

17a A g T C G g C G A G C

G • + + + 48 C T T

G a A G C

C C A A G T CGTAGGGGTT 10 extra arm

| | | G + C C C

20 C + G

20a G + C

20b 30 G + C 40

A + T

C A

T G

T A A

> 2693483 2693566 GCGAGGG TA GCTa AGTCAGGAA-A aAGC G GCGGA CT CAA G - A TCCGC TCC 10 C GtGGG TTCAAAT CCCtC CCCTCGC A CTT **L** Leu (CAA) TTG

1

C T T 76 3'

A

tDNA **L** (CAA) TTG 5' 1 G + C

C + G

G + C 70

A + T

G + C 60

G + C |

16 G + C T A

| 10 T C t C C C A

17 C T G A | A + • + + + A

17a A a T C G G t G G G C

G • + + + 48 C T T

G a A G C

A A - A G T CGTAGGGGTC 10 extra arm

| | | G + C C C

20 C + G

20a G + C

20b 30 G + C 40

A + T

C A

T G

C A A

< 2564858 2564774 GCGTGGG TA GCCa AGCCAGGCCAA cGGC G CAGCG TT GAG G - G CGCTG TCC 10 C gCCGG TTCAAAT CCGGt CCCACGC A TCG **L** Leu (GAG) CTC

1

T C G 76 3'

A

tDNA **L** (GAG) CTC 5' 1 G + C

C + G

G + C 70

T + A

G + C 60

G + C |

16 G + C T A

| 10 T t G G C C A

17 C C G A | A • + + + + A

17a A a C C G g C C G G C

G • + + + 48 C T T

G c G G C

C C A A G T TGTAGAGGTC 10 extra arm

| | | C + G C C

20 A + T

20a G + C

20b 30 C + G 40

G + C

T G

T G

G A G

< 1526350 1526268 GCGCGGG TA GCCa AGT--GGCCAA aGGC G CAGCG CT TAG G - A CGCTG TGG 10 C gCAGG TTCGAAC CCTGt CCCGCGC A TTT **L** Leu (TAG) CTA

1

T T T 76 3'

A

tDNA **L** (TAG) CTA 5' 1 G + C

C + G

G + C 70

C + G

G + C 60

G + C |

16 G + C C A

| 10 T t G T C C A

17 - T G A | A • + + + + G

17a - a C C G g C A G G C

G • + + + 48 C T T

G a G G C

C C A A G T TGTAGACCTT 10 extra arm

| | | C + G G G

20 A + T

20a G + C

20b 30 C + G 40

G + C

C A

T G

T A G

< 2617916 2617832 GCAGGGA TA GCCa AGTCTGGCCAA cGGC G CAGCG TT CAG G - G CGCTG TCT 10 C GCAGG TTCAAAT CCTGC TCCCTGC A CTC **L** Leu (CAG) CTG

1

C T C 76 3'

A

tDNA **L** (CAG) CTG 5' 1 G + C

C + G

A + T 70

G + C

G + C 60

G + C |

16 A + T T A

| 10 T C G T C C A

17 C T G A | A + + + + + A

17a T a C C G G C A G G C

G • + + + 48 C T T

G c G G C

C C A A G T CATAGGAGTC 10 extra arm

| | | C + G C T

20 A + T

20a G + C

20b 30 C + G 40

G + C

T G

T G

C A G

> 458021 458095 GGGCCCC TA GCTC AGTCTGGTC-A GAGC G CtCGG CT CAT A* - A CCGgG TGG T C ATGGG TTCGAAC CCCAT GGGGCCC A TAA I Ile (CAT) ATA

1

T A A 76 3'

A

tDNA i (CAT) ATG 5' 1 G + C

G + C

G + C 70

C + G

C + G 60

C + G |

16 C + G C A

| 10 T T A C C C A

17 C T G A | A + + + + + G

17a T C T C G A T G G G C

G + + + + 48 C T T

G G A G C

T C - A G T T 47

| | | C + G G G

20 t • g

20a C + G

20b 30 G + C 40

G + C

C A

T A

C A T

> 633681 633755 AGCGGGA TG GGAt AGCCAGGAG-A tTCC G CCGGG CT CAT A# - A CCCGG AGA T C GGTAG TTCAAAT CTACC TCCCGCT A TGG M iMet (CAT) ATG

1

T G G 76 3'

A

tDNA m (CAT) ATG 5' 1 A + T

G + C

C + G 70

G + C

G + C 60

G + C |

16 A + T T A

| 10 T C C A T C A

17 C C G A | G + + + + + A

17a A t A G G G G T A G C

G • + + + 48 C T T

G t T C C

A G - A G A T 47

| | | C + G G A

20 C + G

20a G + C

20b 30 G + C 40

G + C

C A

T A

C A T

> 2384839 2384987 GCCCGGG TG GCTt AGCT-GGAC-A tAGC G CCGCA CT CAT A 75 A TGCGG AGA T C GTGGG TTCGGAG CCCAC CCCGGGC A TTC **M** Met (CAT) ATG

1

T T C 76 3'

A

tDNA **M** (CAT) ATG 5' 1 G + C

C + G

C + G 70

C + G

G + C 60

G + C |

16 G + C G A

| 10 T C A C C C G

17 T C G A | G + + + + + G

17a - t T C G G T G G G C

G • + + + 48 C T T

G t A G C

A C - A G A T 47

| | | C + G G A

20 C + G

20a G + C

20b 30 C + G 40

A + T

C A

T A GGGTTTCACAATCGGTGCGGAACGCCTTGGAAGCCTCCGCCCCCCGCGAGGTCATGCCGGCCTCGCACCTGGGAC 75 intron

C A T

> 457852 457924 GCCgCCG TA GCTC AGTT-GGT--A GAGC A CCTCG CT GTT A - A CGAGG TTG T C CCAGG TTCGAGT CCTGG CGGtGGC G CTT **N** Asn (GTT) AAC

1

C T T 76 3'

G

tDNA **N** (GTT) AAC 5' 1 G + C

C + G

C + G 70

g • t

C + G 60

C + G |

16 G + C T G

| 10 T G G T C C A

17 T T G A | A + + + + + G

17a - C T C G C C A G G C

G + + + + 48 C T T

G G A G C

T - - A A T T 47

| | | C + G T G

20 C + G

20a T + A

20b 30 C + G 40

G + C

C A

T A

G T T

< 2358828 2358758 GGGACCG TG gGGt AGT--GGT--A tCCt C TgCCG AT GGG G - T CGGtA GGA - C CTGAG TTCGACT CTCAG CGGTCCC A TAC **P** Pro (GGG) CCC

1

T A C 76 3'

A

tDNA **P** (GGG) CCC 5' 1 G + C

G + C

G + C 70

A + T

C + G 60

C + G |

16 G + C T C

| 10 T G A C T C A

17 - T G A | G + + + + + G

17a - t G G g C T G A G C

G • + + • 48 C T T

G t C C t

T - - A C G - 47

| | | T + A G A

20 g • t

20a C + G

20b 30 C + G 40

G + C

A T

T G

G G G

< 252098 252026 GGGACCG TG gGTt AGCCTGGT--A tACt T CGGGC CT TGG G - T GCCCG TGA - C CCCGG TTCAAAT CCGGG CGGTCCC A TAC **P** Pro (TGG) CCA

1

T A C 76 3'

A

tDNA **P** (TGG) CCA 5' 1 G + C

G + C

G + C 70

A + T

C + G 60

C + G |

16 G + C T A

| 10 T G G G C C A

17 C C G A | G + + + + + A

17a T t T G g C C C G G C

G • + + • 48 C T T

G t A C t

T - - A T T - 47

| | | C + G G A

20 G + C

20a G + C

20b 30 G + C 40

C + G

C T

T G

T G G

> 1836712 1836784 GGGCCGG TG gGGt AGCTTGGT--A tCCt T CGGCC TT CGG G - T GGCCG TAA - C CTCAG TTCGAAT CTGAG CCGGCCC A TAT **P** Pro (CGG) CCG

1

T A T 76 3'

A

tDNA **P** (CGG) CCG 5' 1 G + C

G + C

G + C 70

C + G

C + G 60

G + C |

16 G + C T A

| 10 T G A G T C A

17 T C G A | G + + + + + G

17a T t G G g C T C A G C

G • + + • 48 C T T

G t C C t

T - - A T T - 47

| | | C + G A A

20 G + C

20a G + C

20b 30 C + G 40

C + G

T T

T G

C G G

> 781200 781303 AGTCCCG TG gTGt AGC--GGCCAA tCAt A GCGGC CT TTG G 31 A GCCGC TGA - C GGCGG TTCGAAT CCGCC CGGGACT A CTC **Q** Gln (TTG) CAA

1

C T C 76 3'

A

tDNA **Q** (TTG) CAA 5' 1 A + T

G + C

T + A 70

C + G

C + G 60

C + G |

16 G + C T A

| 10 T C C G C C A

17 - C G A | G + + + + + G

17a - t G T g G G C G G C

G • + + • 48 C T T

G t C A t

C C A A A T - 47

| | | G + C G A

20 C + G

20a G + C

20b 30 G + C 40

C + G

C A

T G ATTGGTACGGTTCATTGCGTGCCGAGAAGAC 31 intron

T T G

> 11661 11733 AGTCCCA TG gGGt AGT--GGCCAA tCCt G TTGCC TT CTG G - G GGCAA CGA - C CCAGG TTCGAAT CCTGG TGGGACT A TTT **Q** Gln (CTG) CAG

1

T T T 76 3'

A

tDNA **Q** (CTG) CAG 5' 1 A + T

G + C

T + A 70

C + G

C + G 60

C + G |

16 A + T T A

| 10 T G G T C C A

17 - T G A | G + + + + + G

17a - t G G g C C A G G C

G • + + • 48 C T T

G t C C t

C C A A G C - 47

| | | T + A G A

20 T + A

20a G + C

20b 30 C + G 40

C + G

T G

T G

C T G

< 452423 452351 GTCCTGA TA gGGt AGT--GGACTA tCCt C CTGGC TT GCG G - A GCCAG GGA - C CGGAG TTCAAAT CTCCG TCAGGAC G TTT **R** Arg (GCG) CGC

1

T T T 76 3'

G

tDNA **R** (GCG) CGC 5' 1 G + C

T + A

C + G 70

C + G

T + A 60

G + C |

16 A + T T A

| 10 T G C C T C A

17 - T G A | A + + + + + A

17a - t G G g C G G A G C

G • + + • 48 C T T

G t C C t

A C T A C G - 47

| | | C + G G A

20 T + A

20a G + C

20b 30 G + C 40

C + G

T A

T G

G C G

< 1307561 1307487 GGGCGCT TA gCTC AGTCTGGAC-A GAGt G CTTGG CT TCG G - A CCAAG TTG C C ACgGG TTCAAAT CCtGT AGCGCCC A TCC **R** Arg (TCG) CGA

1

T C C 76 3'

A

tDNA **R** (TCG) CGA 5' 1 G + C

G + C

G + C 70

C + G

G + C 60

C + G |

16 T + A T A

| 10 T T G t C C A

17 C T G A | A + + • + + A

17a T C T C g A C g G G C

G + + + • 48 C T T

G G A G t

A C - A G T C 47

| | | C + G T G

20 T + A

20a T + A

20b 30 G + C 40

G + C

C A

T G

T C G

> 1692867 1692939 GGgCCCG TA gCTC AGT--GGAC-A GAGt G CTTGG TT CCG G* - A CCAAG ATG C C gCGGG TTCAAAT CCCGt CGGGtCC G TTC **R** Arg (CCG) CGG

1

T T C 76 3'

G

tDNA **R** (CCG) CGG 5' 1 G + C

G + C

g • t 70

C + G

C + G 60

C + G |

16 G + C T A

| 10 T t G C C C A

17 - T G A | A • + + + + A

17a - C T C g g C G G G C

G + + + • 48 C T T

G G A G t

A C - A G A C 47

| | | C + G T G

20 T + A

20a T + A

20b 30 G + C 40

G + C

T A

T G

C C G

> 121223 121296 GgGCGCG TA GCTC AGTC-GGAC-A GAGC G TCGGA CT TCT A - A TCCGA TGG T C gCGGG TTCGAAT CCCGt CGCGCtC G CTC **R** Arg (TCT) AGA

1

C T C 76 3'

G

tDNA **R** (TCT) AGA 5' 1 G + C

g • t

G + C 70

C + G

G + C 60

C + G |

16 G + C T A

| 10 T t G C C C A

17 C T G A | A • + + + + G

17a - C T C G g C G G G C

G + + + + 48 C T T

G G A G C

A C - A G T T 47

| | | T + A G G

20 C + G

20a G + C

20b 30 G + C 40

A + T

C A

T A

T C T

< 1061565 1061493 GGGCGTG TG GCCt AGT--GGAC-A gGGC G AGAGG TT CCT A# - A CCTCT CGA T C gCGGG TTCGAAT CCCGt CACGCCC G CTT **R** Arg (CCT) AGG

1

C T T 76 3'

G

tDNA **R** (CCT) AGG 5' 1 G + C

G + C

G + C 70

C + G

G + C 60

T + A |

16 G + C T A

| 10 T t G C C C A

17 - T G A | G • + + + + G

17a - t C C G g C G G G C

G • + + + 48 C T T

G g G G C

A C - A G C T 47

| | | A + T G A

20 G + C

20a A + T

20b 30 G + C 40

G + C

T A

T A

C C T

< 408423 408343 GCCAGGA TG GCCg AGC--GGT--A aGGC G CACGC CT GGA A - A GCGTG TTC 10 C GgGGG TTCAAAT CCCtC TCCTGGC G CTT **S** Ser (GGA) TCC

1

C T T 76 3'

G

tDNA **S** (GGA) TCC 5' 1 G + C

C + G

C + G 70

A + T

G + C 60

G + C |

16 A + T T A

| 10 T C t C C C A

17 - C G A | G + • + + + A

17a - g C C G G g G G G C

G • + + + 48 C T T

G a G G C

T - - A G T CCTCTGGGAT 10 extra arm

| | | C + G T C

20 A + T

20a C + G

20b 30 G + C 40

C + G

C A

T A

G G A

> 2626534 2626617 GGTGGGA TG GCGg AGT--GGCCTA aCGC G CCTGC CT TGA A - A GCAGG TTT 11 C CTGGG TTCAAAT CCCAG TCCCACC G CAT **S** Ser (TGA) TCA

1

C A T 76 3'

G

tDNA **S** (TGA) TCA 5' 1 G + C

G + C

T + A 70

G + C

G + C 60

G + C |

16 A + T T A

| 10 T G A C C C A

17 - T G A | G + + + + + A

17a - g G C G C T G G G C

G • + + + 48 C T T

G a C G C

C C T A G T CCTCACGGAAT 11 extra arm

| | | C + G T T

20 C + G

20a T + A

20b 30 G + C 40

C + G

C A

T A

T G A

> 575668 575752 GCCGAGG TA GCCT AGCCCGGCC-A AGGC G GTAGA TT CGA A - A TCTAC TGT 11 C GTGAG TTCAAAT CTCAC CCTCGGC G CTT **S** Ser (CGA) TCG

1

C T T 76 3'

G

tDNA **S** (CGA) TCG 5' 1 G + C

C + G

C + G 70

G + C

A + T 60

G + C |

16 G + C T A

| 10 T C A C T C A

17 C C G A | A + + + + + A

17a C T C C G G T G A G C

G + + + + 48 C T T

G A G G C

C C - A G T CCATTCGGACA 11 extra arm

| | | G + C G T

20 T + A

20a A + T

20b 30 G + C 40

A + T

T A

T A

C G A

< 2620371 2620287 GTTGCGG TA GCCa AGCCTGGCCCA aGGC G CTGgG TT GCT A# - A CtCAG TGG 10 C CGGGG TTCGAAT CCCCG CCGCAAC G CTC **S** Ser (GCT) AGC

1

C T C 76 3'

G

tDNA **S** (GCT) AGC 5' 1 G + C

T + A

T + A 70

G + C

C + G 60

G + C |

16 G + C T A

| 10 T G C C C C A

17 C C G A | A + + + + + G

17a T a C C G C G G G G C

G • + + + 48 C T T

G a G G C

C C C A G T CGTCAAGCCC 10 extra arm

| | | C + G G G

20 T + A

20a G + C

20b 30 g • t 40

G + C

T A

T A

G C T

> 1736209 1736280 GCCTgGG TA GCTT AGC--GGT--A AAGC G CGTCC TT GGT A - A GGACG AGA C C CCGGG TTCAAAT CCCGG CCtAGGC T CTT **T** Thr (GGT) ACC

1

C T T 76 3'

T

tDNA **T** (GGT) ACC 5' 1 G + C

C + G

C + G 70

T + A

g • t 60

G + C |

16 G + C T A

| 10 T G G C C C A

17 - C G A | A + + + + + A

17a - T T C G C C G G G C

G + + + + 48 C T T

G A A G C

T - - A G A C 47

| | | C + G G A

20 G + C

20a T + A

20b 30 C + G 40

C + G

T A

T A

G G T

> 2636111 2636184 GCCAGGA GA GCAT GGGC-GGTT-C ATGC A CTCGA CT TGT A - A TCGAG ACT T C GTGGG TTCAAAT CCCAC TCCTGGC T TCT **T** Thr (TGT) ACA

1

T C T 76 3'

T

tDNA **T** (TGT) ACA 5' 1 G + C

C + G

C + G 70

A + T

G + C 60

G + C |

16 A + T T A

| 10 G C A C C C A

17 C G G G | A + + + + + A

17a - T A C G G T G G G C

G + + + + 48 C T T

G A T G C

T T - C A A T 47

| | | C + G C T

20 T + A

20a C + G

20b 30 G + C 40

A + T

C A

T A

T G T

< 840985 840913 GCCGGTG TA GCTC AGTT-GGC--A GAGC G ATTCC TT CGT A - A GGAAT AGG C C GAGGG TTCAAAT CCCTC CACCGGC T TCT **T** Thr (CGT) ACG

1

T C T 76 3'

T

tDNA **T** (CGT) ACG 5' 1 G + C

C + G

C + G 70

G + C

G + C 60

T + A |

16 G + C T A

| 10 T C T C C C A

17 T T G A | A + + + + + A

17a - C T C G G A G G G C

G + + + + 48 C T T

G G A G C

C - - A G A C 47

| | | A + T G G

20 T + A

20a T + A

20b 30 C + G 40

C + G

T A

T A

C G T

> 2328216 2328290 GGGTTGG TG GTCt AGTCTGGTT-A tGAC A CCTCC TT GAC A - T GGAGG AGG C C GGCAG TTCAAAT CTGCC CCAACCC A CTC **V** Val (GAC) GTC

> 2328336 2328410 GGGTTGG TG GTCt AGTCTGGTT-A tGAC A CCTCC TT GAC A - T GGAGG AGG C C GGCAG TTCAAAT CTGCC CCAACCC A CTT **V** Val (GAC) GTC

2

C T T 76 3'

A

tDNA **V** (GAC) GTC 5' 1 G + C

G + C

G + C 70

T + A

T + A 60

G + C |

16 G + C T A

| 10 T C C G T C A

17 C T G A | G + + + + + A

17a T t C T G G G C A G C

G • + + + 48 C T T

G t G A C

T T - A A A C 47

| | | C + G G G

20 C + G

20a T + A

20b 30 C + G 40

C + G

T T

T A

G A C

< 443091 443018 GGGCTCG TG GTCt AGTT-GGTT-A tGAC G CgGCC TT TAC A - A GGCtG AGG T C GGtGG TTCGAAT CCgCC CGAGCCC A TGA **V** Val (TAC) GTA

1

T G A 76 3'

A

tDNA **V** (TAC) GTA 5' 1 G + C

G + C

G + C 70

C + G

T + A 60

C + G |

16 G + C T A

| 10 T C C g C C A

17 T T G A | G + + • + + G

17a - t C T G G G t G G C

G • + + + 48 C T T

G t G A C

T T - A G A T 47

| | | C + G G G

20 g • t

20a G + C

20b 30 C + G 40

C + G

T A

T A

T A C

< 2264075 2264001 GGGTTGG TG GTCt AGCCAGGTT-A tGAC G GCTCC TT CAC A - C GGAGC AGG C C GGCGG TTCGAAT CCGCC CCAACCC A CTT **V** Val (CAC) GTG

1

C T T 76 3'

A

tDNA **V** (CAC) GTG 5' 1 G + C

G + C

G + C 70

T + A

T + A 60

G + C |

16 G + C T A

| 10 T C C G C C A

17 C C G A | G + + + + + G

17a A t C T G G G C G G C

G • + + + 48 C T T

G t G A C

T T - A G A C 47

| | | G + C G G

20 C + G

20a T + A

20b 30 C + G 40

C + G

T C

T A

C A C

> 1165740 1165916 GGGGCtG TG GCCA AGCCCGGC--A TGGC G ACTGA CT CCA G 103 A TCAGT CGA T C GgGGG TTCAAAT CCCtC CgGCCCC A CTC **W** Trp (CCA) TGG

1

C T C 76 3'

A

tDNA **W** (CCA) TGG 5' 1 G + C

G + C

G + C 70

G + C

C + G 60

t • g |

16 G + C T A

| 10 T C t C C C A

17 C C G A | G + • + + + A

17a C A C C G G g G G G C

G + + + + 48 C T T

G T G G C

C - - A G C T 47

| | | A + T G A

20 C + G

20a T + A

20b 30 G + C 40

A + T

C A 103 intron

T G AGGCTTGGCGCCCGGGACGACACTCCAGCTGATATACTGAGCGACCGACTGATCATCGGTCGTGTTGACGACCCTCTGGAGTTCCGAGGTGCCCACCGGAGAT

C C A

> 518441 518514 CCGCTCT TA GCTC AGCCTGGC--A GAGC A GCCGA CT GTA G - A TCGGC TTG T C CCCCG TTCAAAT CGGGG AGAGCGG A TTT **Y** Tyr (GTA) TAC

< 1590452 1590379 CCGCTCT TA GCTC AGCCTGGC--A GAGC A GCCGA CT GTA G - A TCGGC TTG T C CCCCG TTCAAAT CGGGG AGAGCGG A TTT **Y** Tyr (GTA) TAC

2

T T T 76 3'

A

tDNA **Y** (GTA) TAC 5' 1 C + G

C + G

G + C 70

C + G

T + A 60

C + G |

16 T + A T A

| 10 T G G G G C A

17 C C G A | A + + + + + A

17a T C T C G C C C C G C

G + + + + 48 C T T

G G A G C

C - - A A T T 47

| | | G + C T G

20 C + G

20a C + G

20b 30 G + C 40

A + T

C A

T G

G T A

B

**Genetic Code Coverage: Codon / (Anticodon) / AA / tDNAs Nb per Anticodon**

--------------------------------------------------------------------------------------------------

TTT (AAA) Phe **F -** TCT (AGA) Ser **S -** TAT (ATA) Tyr **Y -**  TGT (ACA) Cys **C -**  -

TTC (GAA) Phe **F 2** TCC (GGA) Ser **S 1** TAC (GTA) Tyr **Y 2**  TGC (GCA) Cys **C 1**  1

TTA (TAA) Leu **L 1** TCA (TGA) Ser **S 1**  TAA (TTA) Och *** -** TGA (TCA) Opa *** -**  -

TTG (CAA) Leu **L 1**  TCG (CGA) Ser **S 1** TAG (CTA) Amb *** -** TGG (CCA) Trp **W 1**  1

CTT (AAG) Leu **L -** CCT (AGG) Pro **P -**  CAT (ATG) His **H -**  CGT (ACG) Arg **R -**  -

CTC (GAG) Leu **L 1** CCC (GGG) Pro **P 1**  CAC (GTG) His **H 1**  CGC (GCG) Arg **R 1**  1

CTA (TAG) Leu **L 1** CCA (TGG) Pro **P 1**  CAA (TTG) Gln **Q 1**  CGA (TCG) Arg **R 1**  1

CTG (CAG) Leu **L 1** CCG (CGG) Pro **P 1**  CAG (CTG) Gln **Q 1**  CGG (CCG) Arg **R 1**  1

ATT (AAT) Ile **I -** ACT (AGT) Thr **T -**  AAT (ATT) Asn **N -**  AGT (ACT) Ser **S -**  -

ATC (GAT) Ile **I 1** ACC (GGT) Thr **T 1**  AAC (GTT) Asn **N 1**  AGC (GCT) Ser **S 1**  1

ATA (TAT) Ile **I -**  ACA (TGT) Thr **T 1** AAA (TTT) Lys **K 1**  AGA (TCT) Arg **R 1**  1

ATA (CAT) Ile I **1**

ATG (CAT) Met **M 1** ACG (CGT) Thr **T 1** AAG (CTT) Lys **K 1**  AGG (CCT) Arg **R 1**  1

ATG (CAT) iMet M **1**

GTT (AAC) Val **V -**  GCT (AGC) Ala **A -**  GAT (ATC) Asp **D -**  GGT (ACC) Gly **G -**  -

GTC (GAC) Val **V 2**  GCC (GGC) Ala **A 1**  GAC (GTC) Asp **D 2** GGC (GCC) Gly **G 2**  2

GTA (TAC) Val **V 1** GCA (TGC) Ala **A 2** GAA (TTC) Glu **E 1**  GGA (TCC) Gly **G 1**  1

GTG (CAC) Val **V 1**  GCG (CGC) Ala **A 1**  GAG (CTC) Glu **E 1**  GGG (CCC) Gly **G 1**  1

///
