# Supplementary material for: RNomics and Modomics in the halophilic archaea Haloferax volcanii: identification of RNA modification genes
Source: BMC Genomics. 2008 Oct 9;9:470. doi: 10.1186/1471-2164-9-470 (PMC2584109; doi:10.1186/1471-2164-9-470)
Supplement: Additional File 3 — The hBHBh' structure of the introns in pre-tRNA-Met (CAU), pre-tRNA-Gln (UUG) and pre-tRNA-Trp (CCA) in Haloferax volcanii. [file 1471-2164-9-470-S3.ppt]

## Slide 1
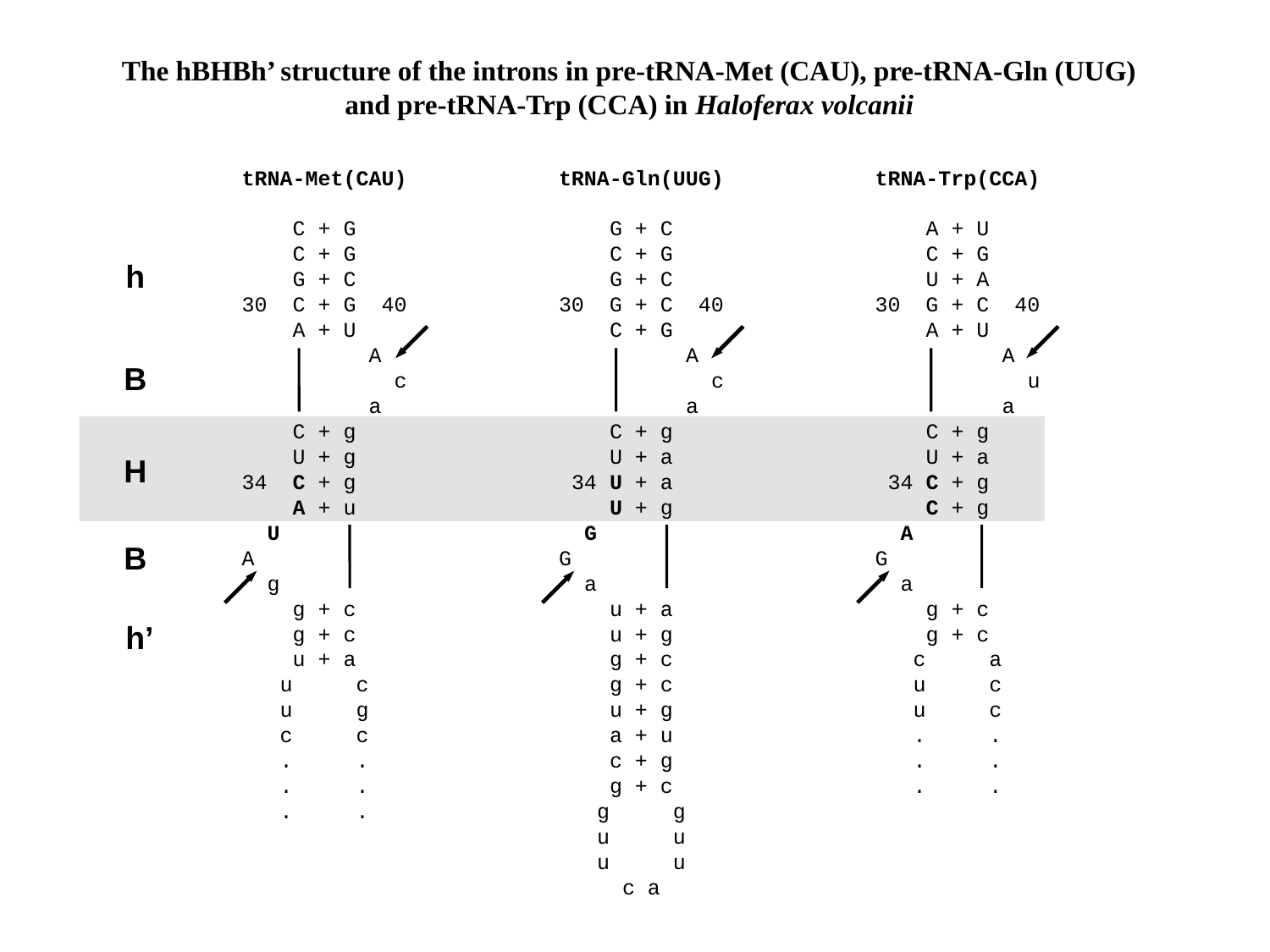

The hBHBh’ structure of the introns in pre-tRNA-Met (CAU), pre-tRNA-Gln (UUG)
and pre-tRNA-Trp (CCA) in Haloferax volcanii
 tRNA-Met(CAU)
 C + G
 C + G
 G + C
 30 C + G 40
 A + U
 A
 c
 a
 C + g
 U + g
 34 C + g
 A + u
 U
 A
 g
 g + c
 g + c
 u + a
 u c
 u g
 c c
 . .
 . .
 . .
 tRNA-Gln(UUG)
 G + C
 C + G
 G + C
 30 G + C 40
 C + G
 A
 c
 a
 C + g
 U + a
 34 U + a
 U + g
 G
 G
 a
 u + a
 u + g
 g + c
 g + c
 u + g
 a + u
 c + g
 g + c
 g g
 u u
 u u
 c a
 tRNA-Trp(CCA)
 A + U
 C + G
 U + A
 30 G + C 40
 A + U
 A
 u
 a
 C + g
 U + a
 34 C + g
 C + g
 A
 G
 a
 g + c
 g + c
 c a
 u c
 u c
 . .
 . .
 . .
h
B
H
B
h’
