# Supplementary material for: RNomics and Modomics in the halophilic archaea Haloferax volcanii: identification of RNA modification genes
Source: BMC Genomics. 2008 Oct 9;9:470. doi: 10.1186/1471-2164-9-470 (PMC2584109; doi:10.1186/1471-2164-9-470)
Supplement: Additional File 4 — sRNAs of Haloferax volcanii predicted to modify Cm34 and Um39 in tRNA-Trp. [file 1471-2164-9-470-S4.doc]

(A)

5' ...GACU**Cm**CAGAGG... 3' (tRNA) 5' ...gauA**Um**CAGU... 3' (tRNA)

|||| |||||| |||| ||||

N**UGAG GUCUCC...AGUAGU** 5' N**UAUA GUCG...AGUAGU** 5'

**C C**

**U U**

**G G**

**A** 3' snoRNA **A** 3' snoRNA

(B)

H. volcanii 1165785-1165869 GCGCCC G **GGACGA** CACUCCA**GCUGAUAU**A **CUGA** GCGACCGACUGAUCAUCGGUCGUGU **UGACGA** C**CCUCUGGAGUU** **CCGA** GGUGC

H. marismortuii 646440-646528 GCGCCU G **GGACGA** CACUCCAAGGGCUGAUAUA **CUGA** GCGGCCGGCUGAUCACCGGUUCGCGA **CGAUGA** CCCUCUGGAGUU **CCGA** GGCGC

H. walsbyi 1627156,1627241 CGCCCG G **UGACGA** UACUCCAGACUGAUAUA **CCGA** GCGAUCAACUGAUCAUUGAUCGCA G **UGACGA** CCCUCUGGGGUA **CCGA** GGCGC

H. NRC-1 770560-770646 GCGCCC G **GGACGA** CGCUCCAGACUGGUAUA **CCGA** GCGGCCGACUGAUCAUCGGUCCGCUG **UGACGA** CCCUCUGGAGUU **CCGA** GGCGC

N. pharaonis 1620497-1620410 CGCCCG G **UGACGA** GACUCCAGACUGAUAUA **CCGA** GCGGACGGCUGAUCACCGCUUCCGCGU **UGACGA** CCCUCUGGAGGA **CCGA** GGCGC

M mazei 2153609-2153534 AUAA A **UGAUGA** ACAACGGGUCUA **CCGA** GUCUUUCCCGACGGGGCAGGACAA **UGAGGA** ACCGUUGGAGCA **CUGA** UAG

M barkeri 1909488-1909413 UAA A **UGAUGA** ACAACGGGUAUA **CUGA** AUCUUUCCCGACGGGGCAAGAUAA **UGAGGA** ACCGUUGGAGCA **CUGA** UAGA

M acetivorans 751022-750948 UAA A **UGAUGA** ACAACGGGUCUA **CCGA** GUCCUUCCCGACGGGGCAGGACAA **UGAGGA** ACCGUUGGAGCA **CUGA** UAG

M. burtonii1 175892-175968 AUAA A **UGAUGA** ACAACGGGUCUA **CUGA** GAUCUGCUCGACGGAGUGGGUCGG **UGAAGA** GCCGUUGGAGCA **CUGA** UGUG

M. thermophila 799970-800045 ACUGG A **UGAUGA** GCAACGGGUCUG **CUGA** GCCAUCCCGACGGGGAUGGUGU **UGAUGA** UCCGUUGGAGCA **CUGA** GAUG

M. marisnigri 1074854-1074792 UUGCAC G **UGAUGA** ACAAUGGGUCUC **CUGA** CGAACGGA **UGAUGA** CCCGUUGGAGCA **CUGA** UGCA

C. Methanoregula 742645-742703 GCAG A **UGAUGA** ACAAUGGGUCUG **CUGA** AAUUGA **UGAUGA** CCCGUUGGAGCA **CUGA** UGCG

M. hungatei 1849259-1849316 GCAG A **UGAUGA** CAAAUGGGUCUU **CUGA** UUUGG **UGAUGA** UCCGUUGGAGCA **CUGA** UGCA

T. kodakarensis 1945728-1945782 GGU A **UGAGGA** CUUGCGGGUUUA **CUGA** UUGG **GGAUGA** GCCUUUGGAGCU **CUGA** CCC

P. horikoshii 597272-597333 GCC A **UGAGGA** UAGGCGGGUUUG **CUGA** CCUCGGGGCG **UGAUGA** ACCUUUGGAGCC **CCGA** GG

P. furiosus 937474-937413 GGCC A **UGAGGA** UAGGCGGGUUUG **CUGA** CCUCGGGGCG **UGAUGA** ACCUUUGGAGCC **CCGA** GGG

P. abyssi 1330583-1330522 GGCC A **UGAGGA** CAGGCGGGUUUG **CUGA** CCUCGGGGCG **UGAUGA** ACCUUUGGAGCC **CCGA** GGG

A. fulgidus 2141684-2141627 GCGGC G **UGAUGA** UUGACGGGUCUG **CUGA** GCGG **UGAUGA** CCCGUUGGAGCU **CUGA** CCCG

T. volcanium GSS1 1440895-1440952 GGAA G **UGAUUA** GAAACAGGGUCAU **CUGA** UUUAA **UGAUGA** GUGACUGGAACU **AUGA** UCC

T. acidophilum 232317-232374 GGAG A **UGAUUA** GAAACAGGGUCAU **CUGA** UUUGA **UGAUGA** GUGACUGGAACU **AUGA** UCC

M. labreanum 1002953-1003012 AGCGU A **UGAUGA** ACUAUCGGGUCUA **CUGA** UUUGA **UGAUGA** CCCGCUGGAGCA **CUGA** UCUA

F. acidarmanus 153165-153116 G **UGAUUA** GCUAAAGGUCGU **CUGA** AUUUA **UGAUGA** AUGACUGGAACG **AUGA**

P. torridus 276518- 276575 GUUU G **UGAUUA** GCGAGAGGGUCAA **CUGA** UUUCA **UGAUGA** UUGACUGGAACU **AUGA** AAC

M. thermautotrophic 1159033-1158971 UUGUCU U **UGAAGA** AAAUGCGCGCU **CUGA** CUACAGUA **UGAUGA** CCAAUUGGAGUA **CUGA** GACAA

M. smithii ATCC 35061 1050209-1050140 UUGUCU U **UGAAGA** AAAUAUACGCGUAA **CUGA** GCAGUAUCCGUUG **UGAUGA** UCAAUUGGAGUA **CUGA** GGCA

P. arsenaticum 1626040-1626103 UUUCC G **UGAUGA** CUUUUUAGUGGGUCCU **CUGA** AUUGG **UGAAGA** UGGUCUGGAGCU **CUGA** UCUAG
