# Supplementary material for: RNomics and Modomics in the halophilic archaea Haloferax volcanii: identification of RNA modification genes
Source: BMC Genomics. 2008 Oct 9;9:470. doi: 10.1186/1471-2164-9-470 (PMC2584109; doi:10.1186/1471-2164-9-470)
Supplement: Additional File 5 — sRNA of Haloferax volcanii predicted to modify Cm34 in tRNA-Met. [file 1471-2164-9-470-S5.doc]

(A)

5' ...CACU**Cm**AUAggg... 3' (tRNA)

|||| ||||||

**GUGAG UAUCCC...** 5'

**C**

**U**

**G**

**A** 3' (sRNA)

(B)

H. volcanii 2163621-2163557 UGGCCG A **UGACGA** CGGCACCGCU **CCGA** ACCGAACUAGGCACUCGGUGA **UGCGGA** G**CCCUAUGAGUG** **CCGA** GGCCU

H. lacusprofundi 605455-605394 G A **UGACGA** UGCACCGCU **CCGA** GCCGCCUCGGCACCGGCGA **UGACGA** GCCCUAUGAGUG **CCGA**

H. sp. NRC-1 1304113-1304039 UGGCCG A **UGACGA** CACCACCGGG **CCGA** CCCGGGGUGGCUCCCGUGGGA **CGACGA** GCCCUAUGAGUG **CCGA** GGCCU

H. marismortui 1688551-1688627 CGGCCG A **UGACGA** CGACUCACUGGG **CCGA** GCCGGGGUGACUCCCUGUUGA **UGACGA** GCCCUAUGAGUG **CCGA** GGCCA

N. pharaonis 2404720-2404794 CGGCCG A **UGACGA** GGCAUCCGGG **CCGA** GUCGGUGUAGGCAGCCGGCAG **CGACGA** GCCCUAUGAGUG **CCGA** GGCCG

H. walsbyi 2150085-2150016 UG A **UGACGA** UGAUACCGUU **CUGA** GUUGGAGUGGCAUCCAUCAA **UGAUGA** ACCCUAUGAGUG **CCGA** GGCCG

M. thermophila 1557381-1557438 GCGCCG A **UGAUGA** CACCAGGUCU **CUGA** AAUG **UGAUGA** GCCCUAUGAGUU **CUGA** GGCGC

M. burtonii 1084094-1084150 CGCCU G **UGAUGA** UGAACUGCA **CUGA** ACCUA **UGAUGA** GCCCUAUGAGUA **CUGA** GGCGA

M. barkeri 73749-73690 GUGCCU G **UGAUGA** AAAAUUAUG **CUGA** UUUCG **UGAUGA** GCCCUAUGAGUU **CUGA** GGCAC

M. mazei 2483728-2483785 GUGCCU G **UGAUGA** AAAAUCUGG **CUGA** UUCCA **UGAUGA** GCCCUAUGAGUU **CUGA** GGCAC

M. acetivorans 1156345-1156401 UGCCU G **UGAUGA** AAAAUCUGG **CUGA** UUUCA **UGAUGA** GCCCUAUGAGUU **CUGA** GGCAC

P. abyssi 1292416-1292359 UGGCGG A **UGAUGA** GCUAGUAAUU **CUGA** GCGA **UGAAGA** GCCCUAUGAGCG **CUGA** CGCCU

P. furiosus 1155598-1155541 CGACGG A **UGAUGA** AAUGUAAAGA **CGGA** AAGA **UGAAGA** GCCCUAUGAGCG **CUGA** CGCCC

P. horikoshii 630832-630888 GGCGG A **UGAUGA** AGGCUAAUUU **CGGA** UUGG **UGAAGA** GCCCUAUGAGCG **CUGA** CGCUA

T. kodakaraensis 287687-287623 GGGCA G **UGAAGA** AUCCAAGACGGG **CUGA** GCAGGGGCGA **UGACGU** ACCCUAUGAGCG **CCGA** GCCCG

M. thermautotrophicus 659833-659771 CCGGC G **UGAUGU** UCGGUUAAUA **CUGA** UUAUGGCCUG **UGAUGA** ACCCUAUGAGCU **CUGA** GAAGU

M. jannaschii 260394-260449 GCCA A **UGAUGA** CGAUUGGCUUUG **CUGA** GUCUG **UGAUGA** ACCGUAUGAGCA **CUGA** GG
