# Supplementary material for: RNomics and Modomics in the halophilic archaea Haloferax volcanii: identification of RNA modification genes
Source: BMC Genomics. 2008 Oct 9;9:470. doi: 10.1186/1471-2164-9-470 (PMC2584109; doi:10.1186/1471-2164-9-470)
Supplement: Additional File 7 — sRNA of Haloferax volcanii predicted to modify Gm1934 (Gm1950) in 23S rRNA. [file 1471-2164-9-470-S7.doc]

(A)

*5' ...*CGGG**Gm**GTAAC... 3' 23S rRNA

||||| |||||

**GCCCC CAUUG**...**AGUAGU** 5'

**C**

**U**

**G**

**A** 3' snoRNA

(B) Alignement of homolog sRNA

H. volcanii 1781375-1781262 GGUGGCG A **UGACGA** AG**GUUACCCCC**A **CUCA** GCCCCUUG **UGAUGA** UAGAUUUCAU **CCGA** GCCACC

H. Marismortuii 1925992-1926058 UGGUGGCG A **UGACGA** GAUGUUACCCCCG **CUGA** GCCCCUUG **UGACGA** UGGACUUUUC **CUGA** GCCACCA

H. salinarum_R1 1925326-1925416 GGCGACG G **UGACGA** GGAGUUACCCCCU **CCGA** GCCCCUUG **UGACGA** GGCCGUUCUC **CCGA** GUCGCCC

H. lacusprofundi ATCC 408532-408468 GGUGGCC A **UGACGA** CGUGUUACCCCCA **CUGA** GCCCCUUG **UGAUGA** GGGUUUUCCA **CCGA** GCCACC

N. pharaonis DSM 2160 341490–341553 GGUGGCG G **UGACGA** CAGUUACCCCCU **CCGA** GCCCCUUG **UGACGA** GGCACUCAUU **CCGA** GCCACC

H. walsbyi DSM 16790 2476861-2476796 UGGUGGCC A **UGAUGA** CAGUUACCCCCA **CUGA** GCCCCUUG **UGAUGU** GGUAUUUUAU **CUGA** GCCACCA

M. marisnigri 657340-657401 GGCGGCAG A **UGAUGA** GAGUUACCCCCA **CUGA** UCCGG **UGAUGA** AGCAGGGGG **CUGA** UGUCGCU

M. vannielii 279348-279440 GCUGU A **UGAUGA** CAGUUACCCCCG **CUGA** CUGGAUUAACUGCUUUAAAGGCUAUUUUUUCGGGA **UGACGA** UAUCAGCACUAU **CUGA** CAAAGCUA

M. maripaludis_C5 729115-729030 CGUUGU A **UGAUGA** CAGUUACCCCCG **CUGA** CUAAAUUCAUGAUUUUCAUGAUUUUGGGA **UGAAGA** CAUCAGCACUAU **CUGA** CACAGC

M. maripaludis_C6 1600143-1600236 CGUUGU A **UGAUGA** CAGUUACCCCCG **CUGA** CUAAAUUCGUGAUUUUCAUGAUUUUAGGA **UGACGA** CAUCAGCACUAU **CUGA** CACAGCUA

M. maripaludis_S2 941692-941605 CGUUGU A **UGAUGA** CAGUUACCCCCG **CUGA** CUAAAUUCGUGAUUUUCAUGAUUUUGGGA **UGAAGA** CAUCAGCACUAU **CUGA** CACAGCUA

M. maripaludis_C7 212028-211941 CGUUGU A **UGAUGA** CAGUUACCCCCG **CUGA** CUAAAUUCGUGAUUUUCAUGAUUUUGGGA **UGAAGA** CAUCAGCACUAU **CUGA** CACAGCUA

M. aeolicus_Nankai-3 860500-860585 CAGCGA A **UGAUGA** CAGUUACCCCCG **CGGA** CUGAAACAAUAUUUAUUUUUGUUUCAGGA **UGACGA** CAUCAGCACUAU **CUGA** CGCAGC

M. labreanum 378814-378879 GGACAG A **UGAUGA** AAGUUACCCCCG **CUGA** ACCGCAAGGUUG **UGAUGA** UGCGUGGUC **CUGA** UGUCCU

M. burtonii 2085602-2085540 GCGGCG A **UGAUGA** GAGUUACCCCGA **CCGA** GCAAAAUA **UGACGA** UAAACUCUAA **CUGA** UGCCGC

M. barkeri 1034452-1034513 UGCGGCG A **UGAUGA** GAGUUACCCCAA **CUGA** GCAAAAGA **UGAUGA** GAACAAUUUC **CUGA** UGCC

M. mazei 1703784-1703721 UGCGGCG A **UGAUGA** AAGUUACCCCGG **CUGA** GCAAAAGA **UGAUGA** GAACAAUUUC **CUGA** UGCCGC

M. acetivorans 161870-161807 UGCGGCG A **UGAUGA** GAGUUACCCCAG **CUGA** GCAAAAGA **UGAUGA** GAACAAUUUC **CUGA** UGCCGC

C. Methanoregula 646118-646064 GGCAA A **UGAUGA** AAGUUACCCCCA **CUGA** CAUUCG **UGAUGA** AGCAGGGGA **CUGA** TG

M. jannaschii 1606357-1606291 ACCUCG A **UGAUGA** AAGUUACCCCCA **CUGA** CCUUUUUGGGA **UGAAGA** AAUCGGCACUGU **CUGA** GAGGU

P. horikoshii 1732055-1731997 AAGCCG A **UGAGGA** UCGUUAGCCACG **CUGA** GGA **UGAUGA** UAAGAGGGUUAG **CCGA** GGCUU

P. abyssi 1755931-1755873 CAGCCG A **UGAGGA** CCGUUAGCCACG **CUGA** GGA **UGAUGA** UAAGAGGGUUAG **CCGA** GGCUU

P. furiosus 1849545-1849603 UGACCG A **UGAGGA** GCGUUAGCCACG **CUGA** AGA **UGAUGA** CAAGAGGGUUAG **CCGA** GGUCU

T. kodakarensis KOD1 1226900-1226841 CGGCCG A **UGACGA** GCGUUAGCCACG **CUGA** CUGA **UGAGGA** AAAGAGGGUUAG **CCGA** GGCCA
