# Supplementary material for: RNomics and Modomics in the halophilic archaea Haloferax volcanii: identification of RNA modification genes
Source: BMC Genomics. 2008 Oct 9;9:470. doi: 10.1186/1471-2164-9-470 (PMC2584109; doi:10.1186/1471-2164-9-470)
Supplement: Additional File 8 — H/ACA sRNA sequences of Haloferax volcanii and some homologous sequences. [file 1471-2164-9-470-S8.doc]

(A)

| **: :**  **C-G**  **C-C**  **5' GTC GAACCCG N(9) ACA 3'**  **||| ||||||**  **3' UGCCAG AΨ UUGGGU 5'**  **23S Ψ2621** |
| --- |

H. vocanii 2499845-249919 TGCGTACCTCAA GTCCCCGGCCGAGTGTTCCCGCTTCGGGAGCG ATGACAGCACGGCGAACCCGGGTGCGCA ATA N(56)

H. lacusprofundi 12758-12566 GACGCGCGCCCTCA GTCCCCGCCCGAGTACTCCCCGATGGGAGCG ATGACAGTGCGGAGAACCCAGGCGCGTC ACA N(41)

H. salinarum 1061525-1061781 TGCACGCCGCT GTCCCCGGCCGAGTACGGCCATGCGGCCGCG ATGACCGCCCGGAGAACCCCGGCGTGCG ACA N(107)

H. marismortuii 317196-316907 GCGTGCCTTTAA TCCCCGGCTGAG ACCCGTCACCGGGACA ATGACGACCCGGAGAACCCGGGCACGCG ACA N(142)

H. walsbyi 2216528-2216709 CACGCACCCTTT GTCCCCGTCCGAGTATTCCTATTATAGGAGCG ATGAACAGACGGCGAACCCGGGTGTGTA ACA N(30)

N. pharaonis 1461830-1462003 TGCACACCATTA GTCCCCGACTGAGTCGCCCCCTTGGGGCAGCG ATGACCGCTCGGAGAACCCAGGTGTGCG ACA N(21)

P. horikoshii 1597482-1597634 GCCCGGCCTTAGCGAGGTCCCCTCGGGAGGCGCCTTCCGCGTCACGGAGTGCCGTGACCGGGGGT AACCCT GGCCGGGC ACA N(0)

P. furiosus 1732773-1732926 GCCCGGCCTCAGCGAGGTCCCCTCGGGAGGTGCCTTCCGCGTCACGGAGTGCCGTGACCGGGGGT AACCCT GGCCGGGC ACA N(0)

P. abyssi 382447-382599 GCCCGGCCTCAGCGAGGTCCCCTCGGTAGGTGCCTTCCGCGTCACGGAGCGCCGTGACCGGGGGT AACCCT GGCCGGGC ACA N(0)

T. kodakarensis 462115-462272 GCCCGGCCTCAGCGAGGTCCCCGCGGGAGGGCCTTCCGCGTCCCGGAGCAC GATGACCGCGGGA AACCCA GGCCGGGC ACA N(5)

I. hospitalis 1066758-1066829

(B)

| **: :**  **C-G**  **C-G**  **C C**  **5' GAGGGUUU CGUUA N(12) ACA 3'**  **||||||| ||||**  **3' CUCCCAGU AΨ CAAU 5'**  **23S Ψ1956** | **: :**  **C C**  **U G**  **U G**  **5’ GGGU CCGUUA N(12) ACA 3’**  **|||| ||||**  **3’ CCCA GΨAUCAAU 5’**  **23S Ψ1958** |
| --- | --- |

H. volcanii CGCCCGGCACGAGGGTTTCCCGGTC GACGCGGCACGCCGCCTCGG GATGA GACCGGCCGTTAGTGTTCTGGGCG ACA

H. lacusprofundi CGCCCGCCACGAGGGTTTCCCGGTT GACGCGGCACGCCGCCGGA GATGA GACCGGTCGTTAGTGTCGCGGGCG ACA

H. salinarum CGCCCGCCACGAGGGTTTCCCGGCC GACAGGGCGTGCCGCCCACG GATGA GGCCGGTCGTTAGTGTTGCGGGCG ACA

H. marismortui CGCCCGGCACGAGGGTTTGCCGGCT GACGTGGCACACCGCCGAG GATGA TGCCGGACGTTAGTGTTCCGGGCGTACA

H. walsbyi CGCCTGACACGAGGGTTTCCCGGCT GATGTGGCATGCCGCCCAAA GATGA TGCCGGTCGTTAGTGTCTCGGGCG ACA

N. pharaonis CGCCCGGCACGAGGGTTAGCCGACT GACCACGGCACGCCGCTTGG GGTGA TGTCGGACGTTAGTGTTCCGGGCGTACA

P. horikoshii GGCCCGTCTG GGTTAGCCCGCCCAATTTTGCCGAGGGCTTA GATGA GGGCGGGTGTTACGGGCGGGCC ACA

P. furiosus GGCCCACCTG GGTTAGCCCGCCTGAGAATGCATACATGCTAC GATGA GGGCGGGTGTTA CGGGTGGGCC ACA

P. abyssi GGCCCGTCT GGTTAGCCCGCCTGATCATGCCGTTGGCTTA GATGA AGGCGGGTGTTACGGGCGGGCT ACA

T. kodakarensis GGCCCGCCAG GGTTAGCCCGCCCAAGGGTGGCGTACGCCTTC GATGA GGGCGGGAGTTACCGGCGGGCC ATA

I. hospitalis GCCCCGCCGC GGGTCAGCCCCGCCGAGTCGGGAATCCCCTCC GATGA GGCGGGGAGTTACCGGCGGGGC ACA
