# Supplementary material for: RNomics and Modomics in the halophilic archaea Haloferax volcanii: identification of RNA modification genes
Source: BMC Genomics. 2008 Oct 9;9:470. doi: 10.1186/1471-2164-9-470 (PMC2584109; doi:10.1186/1471-2164-9-470)
Supplement: Additional File 6 — Representation of C/D box and H/ACA sRNA in archaea. [file 1471-2164-9-470-S6.ppt]

## Slide 1
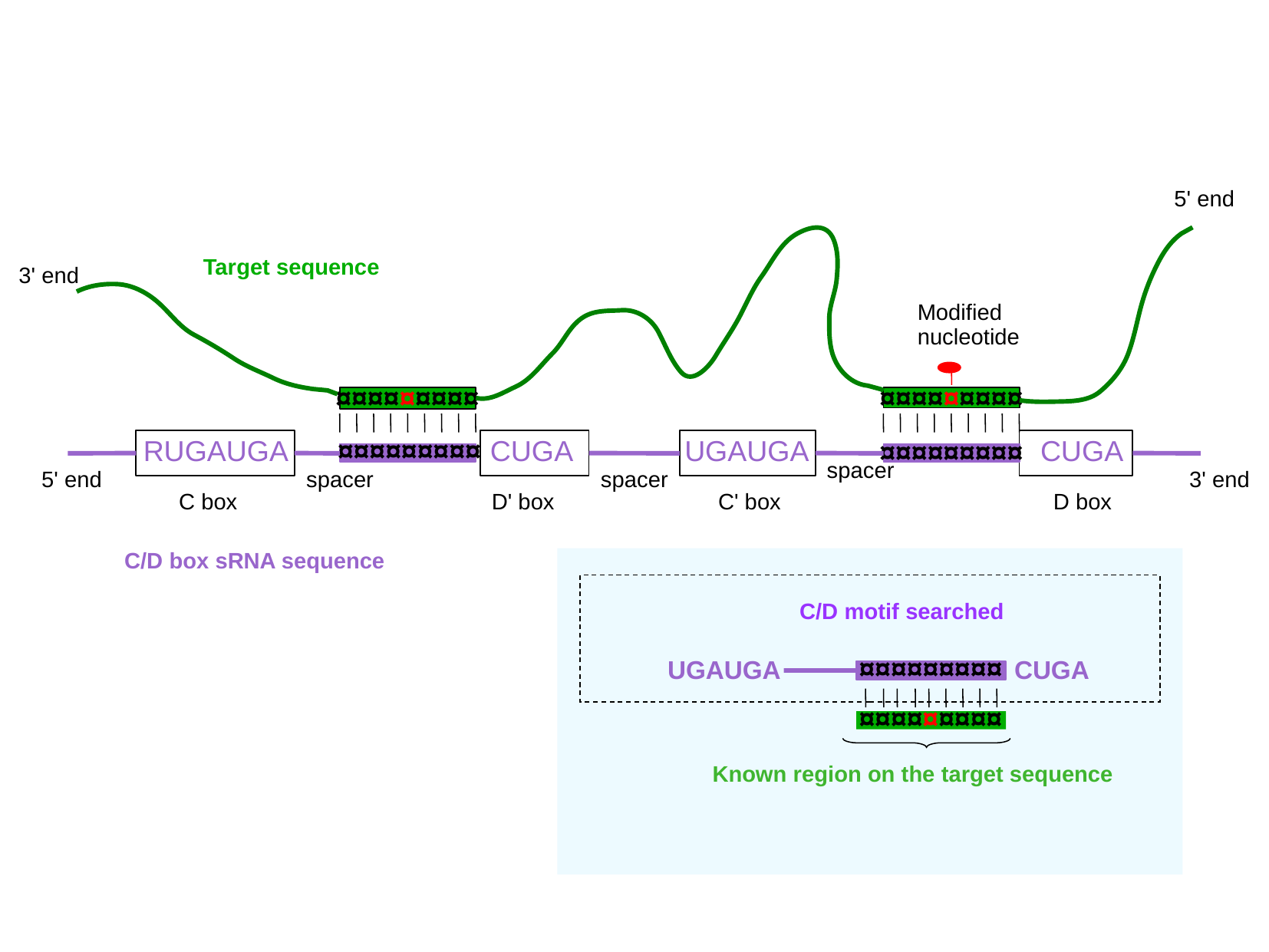

A
5' end
Target sequence
3' end
Modified
nucleotide
¤¤¤¤¤¤¤¤¤
¤¤¤¤¤¤¤¤¤
RUGAUGA CUGA UGAUGA CUGA
¤¤¤¤¤¤¤¤¤
¤¤¤¤¤¤¤¤¤
spacer
5' end
spacer
spacer
3' end
C box
D' box
C' box
D box
C/D box sRNA sequence
 C/D motif searched
UGAUGA CUGA
¤¤¤¤¤¤¤¤¤
¤¤¤¤¤¤¤¤¤
Known region on the target sequence

## Slide 2
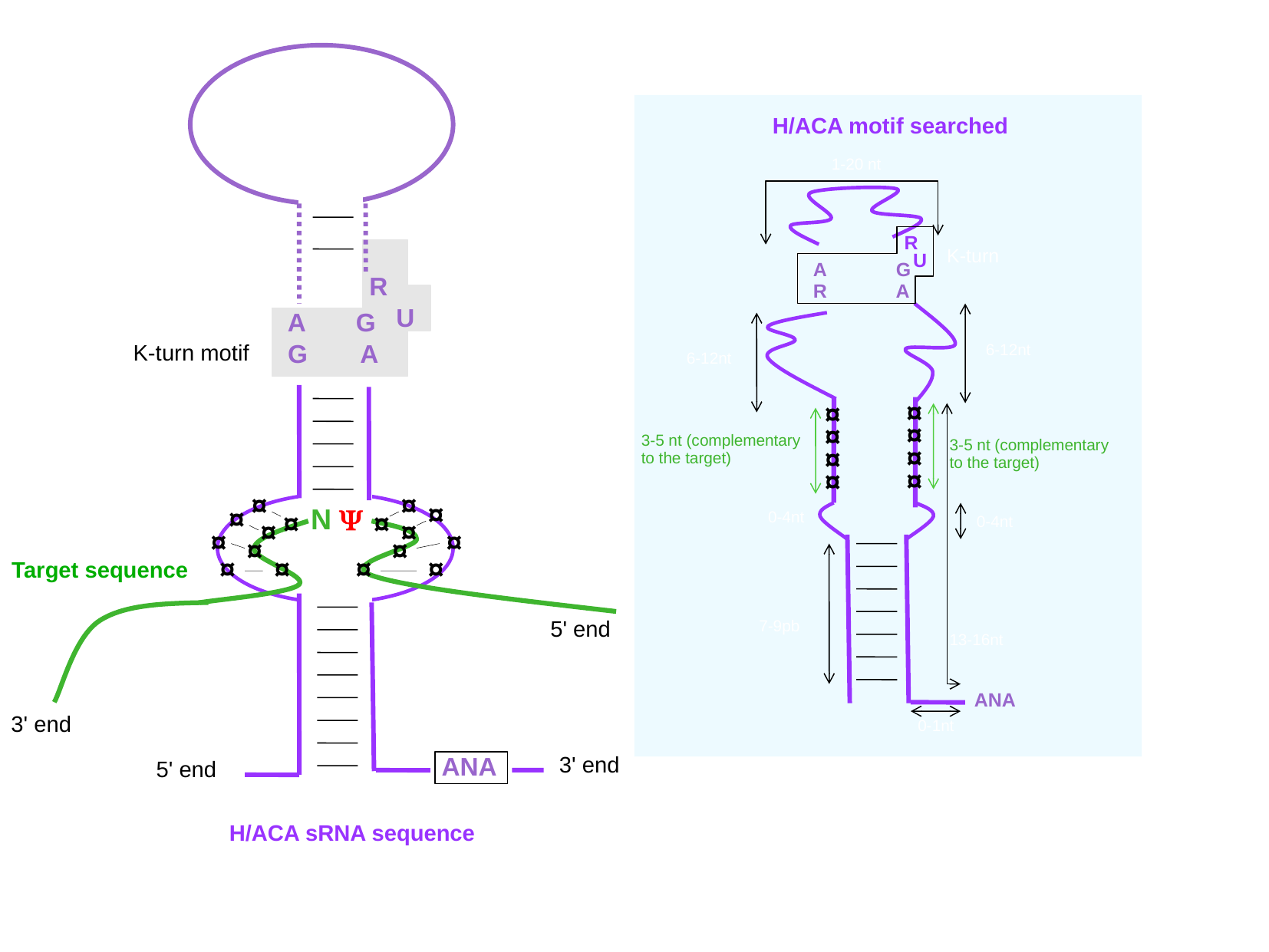

B
H/ACA motif searched
1-20 nt
R
A G
R A
K-turn
U
R
U
A
G
K-turn motif
G
A
6-12nt
6-12nt
¤
¤
¤
¤
3-5 nt (complementary
to the target)
3-5 nt (complementary
to the target)
¤
¤
¤
¤
¤
¤
¤
¤
N 
¤
¤
0-4nt
0-4nt
¤
¤
¤
¤
¤
¤
¤
¤
¤
¤
Target sequence
5' end
7-9pb
13-16nt
 ANA
3' end
0-1nt
ANA
3' end
5' end
H/ACA sRNA sequence
