# Supplementary material for: RNomics and Modomics in the halophilic archaea Haloferax volcanii: identification of RNA modification genes
Source: BMC Genomics. 2008 Oct 9;9:470. doi: 10.1186/1471-2164-9-470 (PMC2584109; doi:10.1186/1471-2164-9-470)
Supplement: Additional File 9 — Legends for Additional files [file 1471-2164-9-470-S9.doc]

##### Additional file legends

**Additional file 1: Sequences of the 52 tDNAs of *Haloferax volcanii* and Genetic Code coverage**

**(A)** The 52 tDNAs are listed according to 1-letter amino acid code under linear and cloverleaf forms. Under the “CAA” heading are listed the 3 nt following nt 73 in the genome. Color code used is: blue, acceptor stem; red, D-stem; cyan, anticodon stem; green, T-stem. Lower case letters are used for mismatches or GT pairing (GU in tRNA). Six tDNAS are present in two copies [note the slight difference in the acceptor stem of the two tDNA-Gly (GCC)]. **(B)** Genetic code coverage (tDNA Usage). Values indicate the number of genes coding for tRNA bearing the anticodon indicated.

**Additional file 2: Sequences of the 41 mature tRNAs + 6 tDNA covering the whole decoding set of *Haloferax volcanii.*** The tRNA sequences were essentially extracted from the work of Ramesh Gupta [28, 29]. They all differ by their anticodons except tRNA-Gly (GCC) which differs in the sequence of their amino acid branch (indicated in bold and underlined). The tDNA sequences in italics, covering the remaining tRNAs harboring a distinct anticodon, are taken from data presented in Supplementary Material SS1 (this paper), using the same color codes for the different domains of the RNA molecules. They all lack the terminal CCA as in the genome of *H. volcanii*. The 6 redundant tDNAs with exactly the same sequence are not given (see text and Supplementary Material SS1). The amino acid are indicated on the left part using the conventional one letter code. The number following the letters corresponds to an arbitrary numbering of the isoacceptor species. Me and Mi correspond to elongator and initiator tRNA-Met respectively. Gray background corresponds to the 3 bases of the anticodon in the middle of the molecule at positions 34-35-36. Position numbering and location of modified nucleotides are indicated in Red and Bold using the conventional one letter code as adopted by [4]. The complete list of the modified nucleotides present in tRNA of *H. volcanii* is given below the list of tRNA sequences Symbol N corresponds to unknown cytidine that is unique to a tRNA-Ile (NAU). Symbol ! corresponds to a yet not clearly defined U derivative, belonging probably to the family of mcm^5^U, eventually thiolated in position 2. See text for details.

**Additional file 3: sRNAs of *Haloferax volcanii* predicted to modify Cm34 and Um39 in tRNA-Trp. (A)** Searched duplex for Cm34 and Um39. **(B)** Alignment of homologous sRNA.. Upper case letters represent mature tRNAs and lower case letters represent intronic region. The color code used is: blue and green, regions involved in the interaction; red, C, C', D and D' box; pink, target regions. The regions involved in the terminal stem are underlined.

**Additional file 4: sRNA of *Haloferax volcanii* predicted to modify Cm34 in tRNA-Met. (A)** Searched duplex for C_m_34. **(B)** Alignment of homologous sRNA sequences. Color code used is: blue, region involved in the interaction; red, C, C', D and D' box; pink, target region. Lower case letters represent the intronic region. Regions involved in the terminal stem are underlined.

**Additional file 5: sRNA of *Haloferax volcanii* predicted to modify Gm1934 (Gm1950) in 23S rRNA. (A)** Searched duplex for Gm1934 (Gm1950). **(B)** Alignment of homologous sRNA. The color code used is: blue, region involved in the interaction; red, C, C', D and D' box; pink, target region.

**SS7: Representation of C/D box and H/ACA sRNA in archaea.** **(A)** C/D box sRNA. The signature used for the research of candidates is given in the dashed rectangle. **(B)** H/ACA box sRNA. The signature used for the research of candidates is given in the blue rectangle.

**SS8: H/ACA sRNA sequences of *Haloferax volcanii* and some homologous sequences predicted to modify Ψ1956-Ψ1958 & Ψ2621 in 23S rRNA**. The color code used is blue for the region involved in the interaction, red for the regions representing K-turn and ACA box. Paired regions are underlined. In this analysis one or two candidate sequences were found, each one corresponding to an hairpin motif able to guide the modification. In all organisms analyzed in this study, we found that the two sequences are located on the same strand and are distant from 0 to 150 nt on the genomic sequence. For each organism, the size of the spacer is given at the end of each sequence contained in the first alignment. (A) 23S rRNA target site for Ψ2621 and alignment of homolog sequences found in this analysis. We did not find a sequence for *Ignococcus hospitalis*. (B) Possible 23S rRNA target sites for respectively Ψ1956 and Ψ1958. Homologous sRNAs are colored in the alignment according to the structure candidate for the Ψ1956 modification.
